# Ubiquitous Polygenicity of Human Complex Traits: Genome-Wide Analysis of 49 Traits in Koreans

**DOI:** 10.1371/journal.pgen.1003355

**Published:** 2013-03-07

**Authors:** Jian Yang, Taeheon Lee, Jaemin Kim, Myeong-Chan Cho, Bok-Ghee Han, Jong-Young Lee, Hyun-Jeong Lee, Seoae Cho, Heebal Kim

**Affiliations:** 1 University of Queensland Diamantina Institute, University of Queensland, Princess Alexandra Hospital, Brisbane, Queensland, Australia; 2 Department of Agricultural Biotechnology, Seoul National University, Seoul, Korea; 3 Interdisciplinary Program in Bioinformatics, Seoul National University, Seoul, Korea; 4 Center for Genome Science, Korea National Institute of Health, Osong Health Technology, Chungcheongbuk-do, Korea; 5 Division of Animal Genomics and Bioinformatics, National Institute of Animal Science, Rural Development Administration, Suwon, Korea; 6 C&K Genomics, Seoul, Korea; Vanderbilt University, United States of America

## Abstract

Recent studies in population of European ancestry have shown that 30%∼50% of heritability for human complex traits such as height and body mass index, and common diseases such as schizophrenia and rheumatoid arthritis, can be captured by common SNPs and that genetic variation attributed to chromosomes are in proportion to their length. Using genome-wide estimation and partitioning approaches, we analysed 49 human quantitative traits, many of which are relevant to human diseases, in 7,170 unrelated Korean individuals genotyped on 326,262 SNPs. For 43 of the 49 traits, we estimated a nominally significant (*P*<0.05) proportion of variance explained by all SNPs on the Affymetrix 5.0 genotyping array (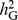). On average across 47 of the 49 traits for which the estimate of 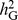 is non-zero, common SNPs explain approximately one-third (range of 7.8% to 76.8%) of narrow sense heritability.

The estimate of 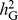 is highly correlated with the proportion of SNPs with association *P*<0.031 (*r* ^2^ = 0.92). Longer genomic segments tend to explain more phenotypic variation, with a correlation of 0.78 between the estimate of variance explained by individual chromosomes and their physical length, and 1% of the genome explains approximately 1% of the genetic variance. Despite the fact that there are a few SNPs with large effects for some traits, these results suggest that polygenicity is ubiquitous for most human complex traits and that a substantial proportion of the “missing heritability” is captured by common SNPs.

## Author Summary

The “missing heritability” problem has been intensely debated for the last few years. Possible explanations include the existence of many genetic variants each with a small effect, rare variants with large effects, and heritability being over-estimated. Previous studies using whole-genome estimation have demonstrated that for human complex traits such as height, body mass index, and intelligence, a large portion of the heritability can be captured by all the common SNPs on the current genotyping arrays. These studies, however, were all concentrated only on a few traits. In this study, we analysed 49 quantitative traits in a sample of ∼7,000 unrelated Korean individuals. We found that, on average over all the traits, common SNPs on the Affymetrix 5.0 genotyping array explain approximately a third of the heritability, that genetic variants are widely distributed across the whole genome with longer chromosomes explaining more phenotypic variation, and that approximately any 1% of the genome explains 1% of the heritability. Despite examples where a few variants explain a substantial amount of variation, all these results are consistent with polygenicity being ubiquitous for most complex traits.

## Introduction

The five years wave of genome-wide association studies (GWAS) has uncovered thousands of single nucleotide polymorphisms (SNPs) to be associated with hundreds of human complex traits including common diseases [1], [2]. Yet, for most complex traits, the gap between the proportion of phenotypic variance accounted for by the top SNPs that reached genome-wide significance level in GWAS and the heritability estimated from pedigree analyses remains unexplained [3]. This was called the “missing heritability” problem [4], explanations to which have been debated in the field [3]. Taking height and BMI for example, well-powered studies with a discovery sample of over 100,000 individuals have identified 180 and 32 loci to be associated with height [5] and BMI [6], which explain ∼10% and ∼1.5% of variance for height and BMI, respectively, while the heritability was estimated to be ∼80% for height [7] and 40∼60% for BMI [8], [9]. On the other hand, however, recent studies using whole-genome estimation approaches have demonstrated that a large proportion of heritability for height [10], [11], body mass index (BMI) [11], schizophrenia [12] and rheumatoid arthritis (RA) [13] can be captured by all the common SNPs on the current genotyping arrays, which implies that there are a large number of variants each with an effect too small to pass the stringent genome-wide significance level. It could be argued that the evidence from these whole-genome estimation analyses are for the traits that are known to be highly polygenic and therefore are not representative for most human complex traits. Therefore, it remains unclear whether polygenic inheritance is a general phenomenon for most human complex traits or a unique feature for a particular group of traits such as height and BMI. There has been evidence from a review of a number of GWAS that more variants have been identified with increased sample size [2], consistent with a pattern of polygenic inheritance for most common diseases and complex traits. In this study, using the whole-genome estimation and partitioning approaches [10], [11], [14], we directly estimated the proportion of phenotypic variance explained by the common SNPs all together on a genotyping array for a range of quantitative traits in a large homogenous sample of Koreans. We demonstrated by a number of different analyses that polygenic inheritance is likely to be ubiquitous for most human complex traits.

## Results

We used the data from the Korea Association Resource (KARE) project [15]. The KARE cohort consists of 10,038 individuals recruited from two different sites in South Korea, genotyped at 500,568 SNPs on Affymetrix Human SNP array 5.0. There were 7,170 unrelated individuals and 326,262 autosomal SNPs after quality controls (Materials & Methods). We show by principal component analysis that all the individuals are of eastern Asian ancestry (Figure S1). All the individuals were measured for 49 quantitative traits, which are related to obesity, blood pressure, hyperglycemia, diabetes, liver functions, lung functions, and kidney functions (Table S1). The phenotypic correlations between pairwise traits are visualized in Figure S2, with traits within the same classification groups being more correlated than between groups.

We then estimated the proportion of variance explained by fitting all the SNPs in a mixed linear model for each of the 49 traits (Materials & Methods). In general, there was a substantial amount of variance explained by all SNPs on the Affymetrix 5.0 genotyping array (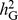) for most traits with a mean of 12.8% (a range from 0 to 31.6%) across all the 49 traits (Table 1). For 47 of the 49 traits, the estimate of 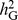 was non-zero, 43 of which reached the nominal significance level (likelihood ratio test *P*<0.05) and 26 of which reached experimental-wise significance level after Bonferroni correction for multiple traits (likelihood ratio test *P*<0.001) [14]. We compared the estimates of 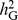 with the narrow-sense heritability (*h* ^2^) estimated from pedigree analyses in the literature (Table S2), and observed a significant trend (*P* = 0.017) that traits with a higher estimate of *h* ^2^ were more likely to have a larger estimate of 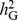 (Figure S3) and that all the common SNPs explain approximately 33.3% (a range from 7.8% to 76.8%) of the narrow-sense heritability, despite that the estimates of *h* ^2^ were from various different studies, usually with large standard errors and mostly in samples of European ancestry. In contrast, when we performed a genome-wide association (GWA) analysis in the same sample, we identified genome-wide significant (*P*<5×10^−8^) SNPs for 25 of the 49 traits. On average across the 25 traits, the top associated SNPs from GWA analyses explained only 1.5% (range of 0.5% to 3.8%) of phenotypic variance (Table S2), nearly 10-fold smaller than the estimate of 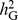, suggesting there are many SNPs remaining undetected because of the lack of statistical power. In addition, we estimated the variance explained by all the SNPs imputed to HapMap2 CHB and JPT panels (Materials & Methods and Table S2). The estimate of 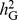 averaged across all the traits using imputed data (13.8%) was slightly higher than that using genotyped data (12.8%).

**Table 1:** Estimates of variance explained by all SNPs for the 49 traits.

Group	Trait	*n*	a 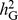 (SE)	*P*
	Height	7170	0.316 (0.042)	2.1e-15
**Obesity**	BMI	7168	0.147 (0.041)	1.1e-04
	Waist	7163	0.105 (0.040)	4.1e-03
	Hip	7160	0.126 (0.040)	7.0e-04
	WHR	7160	0.082 (0.040)	2.0e-02
	Weight	7168	0.161 (0.040)	1.8e-05
	SUB	7138	0.203 (0.041)	1.0e-07
	SUP	6570	0.089 (0.043)	1.7e-02
**Blood Pressure**	SBP0	7170	0.221 (0.041)	1.1e-08
	SBP	7169	0.250 (0.041)	5.8e-11
	DBP0	7169	0.217 (0.041)	3.7e-08
	DBP	7170	0.171 (0.041)	6.7e-06
	Pulse	7162	0.119 (0.041)	1.6e-03
**BMD**	DS	6753	0.135 (0.043)	6.0e-04
	MS	6771	0.107 (0.042)	4.3e-03
**Lipids**	HDL	7169	0.172 (0.041)	8.5e-06
	TCHL	7169	0.156 (0.040)	2.3e-05
	TG	7169	0.216 (0.041)	1.5e-08
	LDL	6963	0.134 (0.041)	3.8e-04
	NONHDL	7169	0.157 (0.040)	1.9e-05
	THDL	7169	0.162 (0.040)	1.4e-05
**Diabetes**	GLU0	7006	0.112 (0.041)	2.9e-03
	GLU60	6824	0.104 (0.043)	7.2e-03
	GLU120	6830	0.118 (0.042)	1.5e-03
	INS0	7007	0.000 (0.040)	5.0e-01
	INS60	6823	0.074 (0.042)	3.9e-02
	INS120	6824	0.144 (0.043)	3.8e-04
	HBA1C	7168	0.126 (0.040)	5.8e-04
	HOMA	7006	0.000 (0.040)	5.0e-01
**Blood CellCount**	WBC	7169	0.162 (0.041)	2.3e-05
	RBC	7169	0.186 (0.041)	1.1e-06
	PLAT	7169	0.196 (0.041)	3.5e-07
	HCT	7169	0.091 (0.040)	9.6e-03
**Blood Ions**	SONA	7169	0.063 (0.039)	4.7e-02
	POTA	7169	0.047 (0.039)	1.2e-01
	CHL	7169	0.113 (0.039)	9.1e-04
**Liver Functions**	CRP	7168	0.109 (0.039)	1.1e-03
	HB	7169	0.064 (0.039)	4.9e-02
	AST	7169	0.072 (0.040)	3.0e-02
	ALT	7169	0.146 (0.040)	7.4e-05
	RGTP	7169	0.109 (0.040)	2.9e-03
**LungFunctions**	SP1	7009	0.226 (0.043)	2.1e-08
	SP2	7007	0.134 (0.041)	4.2e-04
	SP3	7011	0.148 (0.041)	1.0e-04
**Kidney Functions**	RENIN	7169	0.076 (0.039)	2.3e-02
	Bun	7169	0.102 (0.040)	4.7e-03
	Creatine	7169	0.048 (0.040)	1.1e-01
	SG	7147	0.034 (0.039)	1.9e-01
	pH	7147	0.039 (0.040)	1.7e-01

a Estimate of variance explained by all SNPs with its standard error given in the parentheses. A full version of this table can be found in Table S2.

**Figure 1:**
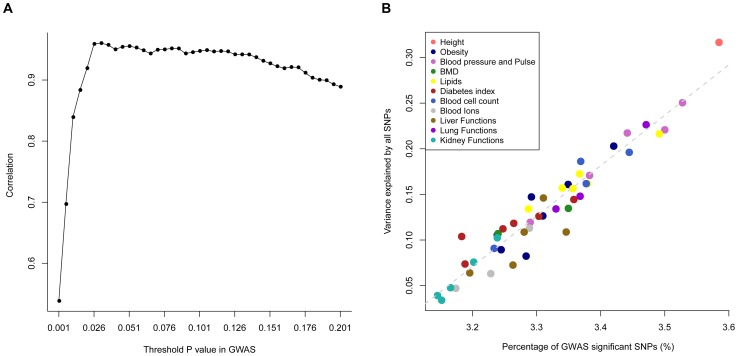
Estimate of variance explained by all SNPs ( 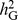) versus proportion of GWAS significant SNPs. The proportion of GWAS significant SNPs (*θ*_P_) is defined as the proportion of SNPs that passed a threshold *P* value (e.g. 0.01) in GWAS. Panel **A**): correlations (*r*) between *θ*_P_ and 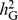 across 47 traits (all traits except INS0 and HOMA) for a range of threshold p-values. The maximum *r* value (*r*_max_ = 0.960) is at a threshold p-value of 0.031. Panel **B**): estimates of 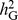 against *θ*_P_ at p-value of 0.031 for the 47 traits.

We calculated the proportion of SNPs with p-values that passed a threshold p-value in a GWA analysis (*θ*_P_) for each trait. We calculated *θ*_P_ for a range of threshold p-values and plotted them against the expected values under the null hypothesis of no association (i.e. the threshold p-values) (Figure S4). This plot is an analogue to the QQ plot. The averaged *θ*_P_ over all the traits started deviating from the expected value when the threshold p-value became small (Figure S4A) and such deviation varied across traits (Figure S4B). The question is whether a trait that shows a larger value of *θ*_P_ will also tend to have a larger estimate of 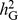. We then correlated *θ*_P_ with the estimates of 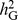 across all the traits for a threshold p-value and calculated such correlations for a range of threshold p-values, from 0.001 to 0.201 by 0.05. We found a maximum of squared correlation of 0.923 at the threshold p-value of 0.031 (Figure 1), meaning that traits that have more proportion of SNPs passed a significance level in GWAS also have more proportion of phenotypic variance explained by all SNPs. It should be noted that the threshold p-value at which the maximum correlation between the estimate of 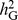 and *θ*_P_ was found depends on sample size. This analysis is an alternative way to demonstrate the equivalence between GWAS and the whole-genome estimation analysis as implemented in GCTA. Although the whole-genome estimation approach estimates the variance explained by all SNPs regardless of individual SNP-trait associations, the estimate of 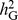 is actually mainly attributed to SNPs that show stronger evidence for association with the trait, e.g. ∼92% of the estimate of 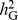 could be determined by SNPs with association p-values<0.031 given the sample size of ∼7,000 in this study. These results also suggest that there are many common variants associated with the traits at nominally significant level (*P*<0.05) but their effect sizes are too small to be genome-wide significant (*P*<5×10^−8^).

**Figure 2:**
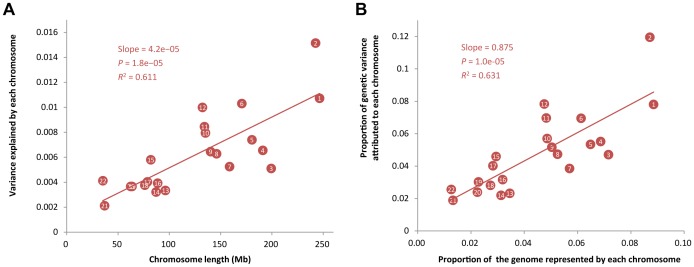
Proportion of variance attributed to each chromosome averaged across 47 traits against chromosome length. In panel A), shown on the y-axis is the averaged estimate of variance explained by each chromosome (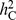) across all the traits, except INS0 and HOMA, for which the estimates of variance explained by all SNPs (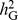) are zero. In panel B), the estimate of 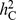 is weighted by for each trait, i.e. 
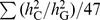
, and the length of each chromosome is divided by the total length of the genome, where the intercept (0.008, SE = 0.007) is not significantly different from zero (*P* = 0.289) and the slope (0.875, SE = 0.150) is not significantly different from 1, which is not significantly different from 1 (*P* = 0.413).

**Figure 3:**
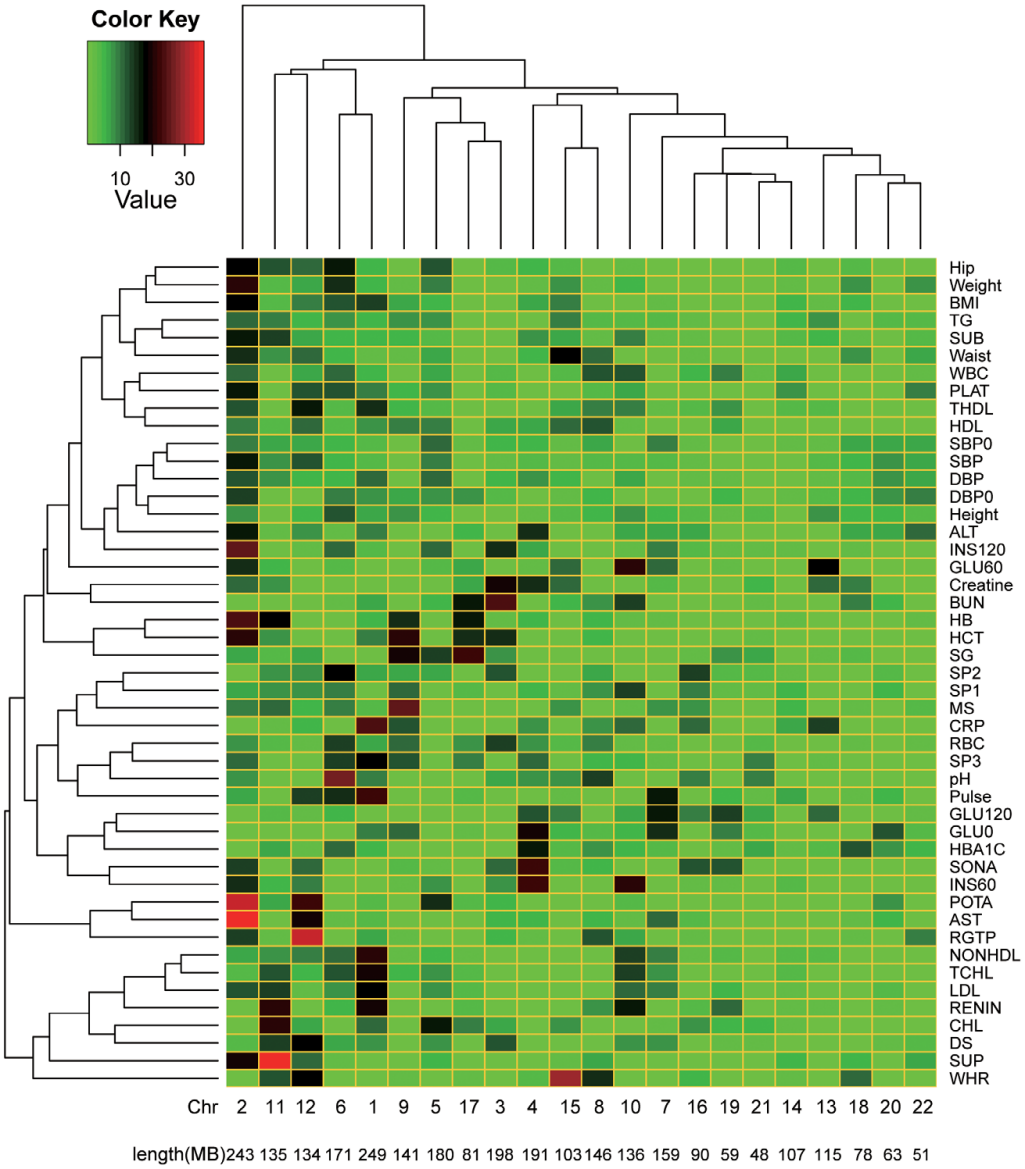
Heatmap of the proportions of variance explained attributed to individual chromosomes for 47 traits. On the y-axis is the variance explained by each chromosome (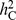) weighted by the total variance explained by all SNPs (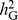) and averaged across all traits, except INS0 and HOMA, for which the estimates of are zero. The estimates of 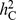 and the traits were clustered by the hierarchical clustering approach and the heatmap plot was generated by the gplots package in R.

Using the same method as above but allowing to fit multiple genetic components simultaneously in the model (Materials & Methods), we then partitioned 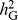 into the contributions of individual chromosomes for all the 49 traits (Table S3) except HOMA and INS0 for which the estimates of 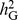 were zero (Table 1), and plotted the estimate of variance explained by each chromosome (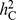) against chromosome length (*L*_C_) for each trait. We did not observe a linear correlation between 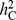 and *L*_C_ for any particular traits (Figure S5) as strong as that shown in the previous studies for height [11] and schizophrenia [12]. The squared correlation between 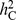 and *L*_C_ was from 0.00 to 0.48 with a mean of 0.15 and a standard deviation of 0.12. This result is not unexpected because the sample size of this study is smaller than that of the previous analysis so that 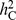 in our analysis were estimated with larger sampling errors. We then averaged the estimates of 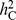 over all the traits to reduce the sampling error variance and found that the averaged estimate of 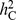 was strongly correlated with *L*_C_ with a correlation of 0.78 (Figure 2A). We show by hierarchical cluster analysis that the correlation between averaged 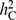 and *L*_C_ was not driven by a few traits (Figure 3) and by randomly sampling the same number of SNPs from each chromosome that it was also not due to longer chromosomes having more SNPs (Figure S6). We also demonstrate that the estimates of 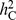 on longer chromosomes were more variable than those on shorter chromosomes (Figure S7). We further took the weighted average of the estimates of 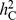 across traits by 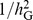, which is defined as the proportion of genetic variance attributed to each chromosome, and plotted it against the proportion of the genome represented by each chromosome (*L*_C_/*L*, with *L* being the total length of the genome) (Figure 2B). The regression slope of the proportion of the genetic variance attributed to each chromosome on the proportion of the genome represented by each chromosome was 0.875 with a standard error (SE) of 0.150 which was not significantly different from 1 (*P* = 0.413), and the intercept was 0.008 (SE = 0.007) which was not significantly different from zero (*P* = 0.289), suggesting that on average 1% of the genome approximately explains 1% of the genetic variance. Despite that there are SNPs with large effects for some traits (Figure S8), all these results are consistent with that many genetic variants each with a small effect widely spread across the whole genome.

**Figure 4:**
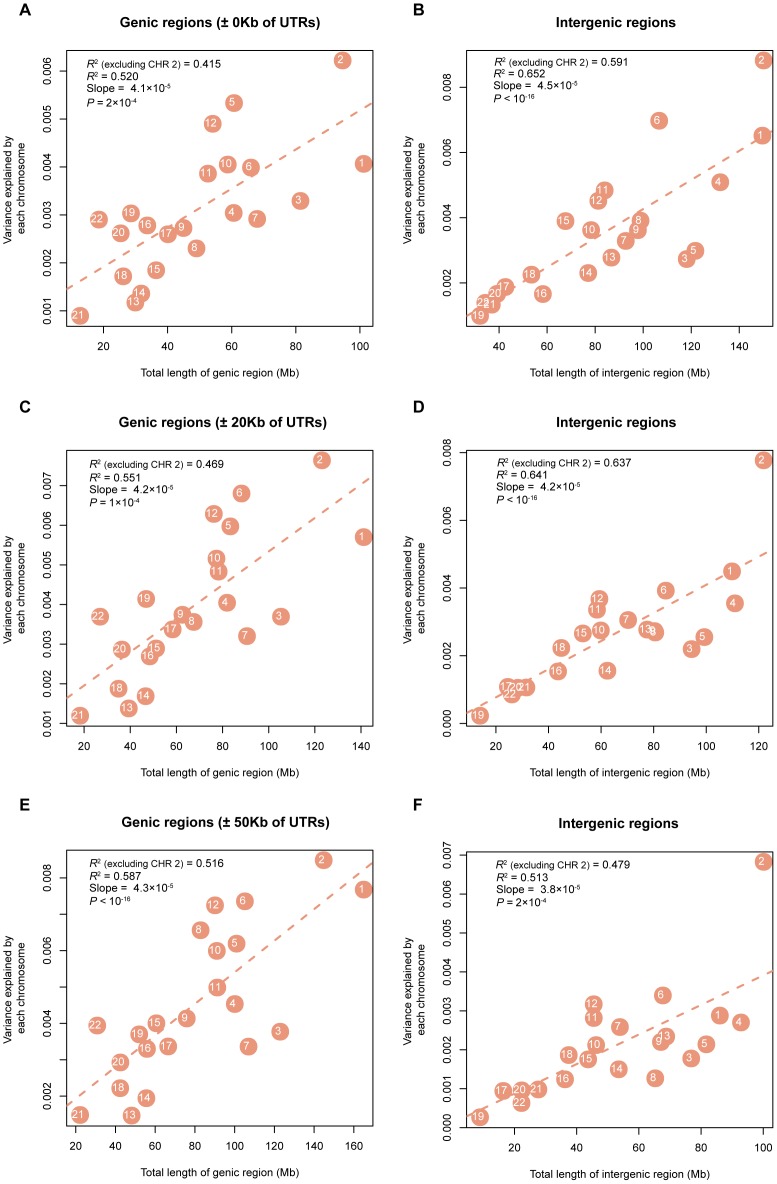
Estimates of the variance explained by all SNPs in genic (intergenic) regions averaged across 47 traits (all traits except INS0 and HOMA) against length of genic (intergenic) DNA. Shown on panels A), C) and E) are the results for the genic SNPs, and shown on panels B), D) and F) are the results for intergenic SNPs, under the three definitions of genic regions, ±0 Kb, ±20 Kb and ±50 Kb of UTRs, respectively.

In addition, we partitioned 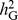 into the contributions of genic (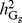) and intergenic (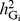) regions of the whole genome (Materials & Methods) and averaged the estimates of 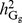 and 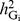 across all the traits. The result shows that SNPs in genic regions explain disproportionally more variation than those in intergenic regions (Table S4). We further estimated the variance explained by the genic (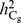) and intergenic (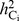) regions of each chromosome and again averaged the estimates of 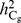 and 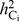 across all traits. The numbers of genic and intergenic SNPs on each chromosome are presented in Table S5. We show that the variance explained by the genic (intergenic) regions on each chromosome is also proportional to the total length of the genic (intergenic) regions (Figure 4).

## Discussion

Previous studies using the whole-genome estimation approach [10], [14] have shown that common SNPs explain a large proportion of heritability for traits and diseases such as height [10], [11], BMI [11], cognition ability [16], [17], rheumatoid arthritis [13] and schizophrenia [12]. The reason why GWAS have not yet identified all the common SNPs that explain this amount of variation is mainly because there are many of them each with an effect too small to pass the stringent genome-wide significance level. However, each of these studies focused only on one or a few diseases or traits. We estimated and partitioned the genetic variance that tagged by all common SNPs for 49 traits in an eastern Asian population and showed by a number of analyses that polygenic inheritance is ubiquitous for most human complex traits.

The estimates of 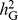 for 6 traits, however, were not different from zero at the nominal significance level (0.05) and the estimates for two insulin related traits INS0 (fasting blood insulin level) and HOMA (homoeostasis model assessment for insulin resistance) were constrained at zero in the analysis because the estimates were converged at small negative values during the estimation process. It does not necessarily mean that common SNPs do not explain any genetic variance for INS0 and HOMA. It could mean that 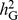 for the two traits are small and their estimates approached zero just because of random sampling. For example, if the true parameter of 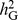 for a trait is 0.05, given a SE of 0.04 (similar magnitude as those presented in Table 1), the probability of getting a zero estimate of 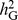 is approximately 0.11, meaning that it is not surprising to observe a few zero estimates from an analysis of 49 estimates if the true parameters of 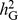 for these traits have a spectrum from moderate to small values.

The estimate of 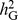 for height was 31.6% (SE = 4.6%), which was smaller than the estimate from a study in Australians (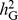 = 44.9%, SE = 8.3%) [10] but not statistically significant (*P* = 0.161), and was significantly (*P* = 0.015) smaller than the estimate from another study in European Americans (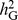 = 44.8%, SE = 2.9%) [11]. There could be two possible reasons: 1) there is a difference in heritability for height between Koreans and Europeans and 2) the tagging of Affymetrix 5.0 array is not as good as the later version Affymetrix 6.0 and the Illumina HumanCNV370 arrays used in the previous studies in Europeans. The estimate for BMI (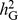 = 14.7%, SE = 4.1%) was also slightly smaller than that in European Americans (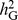 = 16.5%, SE = 2.9%) [11] but the difference was not significant (*P* = 0.741). We estimated the narrow-sense heritability for 11 traits by from a family study in Koreans (Text S1 and Table S6). The estimate of heritability either for height (*h* ^2^ = 0.744, SE = 0.048) or for BMI (*h* ^2^ = 0.478, SE = 0.057) in Koreans was comparable to that estimated in Europeans. We then estimated the variance explained by all SNPs on Affymetrix 5.0 array in the sample of 11,586 unrelated European Americans as used in [11] (Text S1). The estimate of variance explained by all SNPs on Affymetrix 5.0 array in European Americans was 0.394 (SE = 0.027) for height, which was not significantly different from that estimated in this study (*P* = 0.118). Therefore, the difference between the estimate of 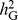 in this study and in previous studies is partly due to the use of different types of SNP genotyping arrays and partly due to sampling error.

It is demonstrated by the genome partitioning analysis that there was a strong linear relationship between the estimates of variance explained by individual chromosomes and chromosome length (Figure 2). The correlation between variance explained and DNA length was stronger in the intergenic regions than that in the genic regions if we define the genic region as ±0 Kb or ±20 Kb of UTRs, while it was stronger in the genic regions than that in the intergenic regions if we define the genic region as ±50 Kb of UTRs (Figure 4). We show by a number of analyses that the result was driven neither by the difference between the number of SNPs in genic regions and in intergenic regions nor by the difference in MAF distribution between genic and intergeinc SNPs (Text S2). If trait-associated genetic variants are enriched in functional elements such as introns and UTRs and diluted in exons, the relationship between the variance explain and DNA length will be attenuated in the genic region. However, this could also be just due to sampling. The sampling variance of a regression *R* ^2^ is approximately 4*ρ* ^2^(1−*ρ* ^2^)/*N* where E(*R* ^2^) = *ρ* ^2^ and *N* is number of observations (number of chromosomes in this case). Given *ρ* ^2^ = 0.5 and *N* = 22, the SE of the regression *R* ^2^ is ∼0.2. Therefore, the difference between the correlation (between the variance explained and DNA length) in genic regions and that in intergenic regions is unlikely to be significant. In addition, in the partitioning analysis of intergenic regions, chromosome 2 seems to be an outlier (Figure 4). For example, for the definition of genic region of ±50 Kb, the variance explained by the intergenic regions on chromosome 2 averaged across 47 traits was 0.68% (SE = ∼0.16%), which was 0.25% larger than the expected value from the fitted line. Given the SE of ∼0.16%, the difference was, however, not greater than what we would expect by chance (*P* = 0.118).

Moreover, we attempted to investigate the enrichment of genetic variants in genes involved in biological pathways. For any particular trait, there are a number of biological pathways that are important to the trait development. We chose the well-known insulin signal transduction pathway as an example to demonstrate the use of GCTA to partition the genetic variance based on functional annotations. We took SNPs that are ±20 kb away from 103 genes that are involved in insulin signaling pathway. There were 955 SNPs which covered ∼0.45% of the genome. We then performed the genome partitioning analysis to decompose 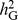 into two components, i.e. the contribution of the genes involved in insulin pathway and that of the rest of the genome for 11 lipids and diabetes related traits. As shown in Table S7, we did not find any evidence that genes involved in insulin pathway explained disproportionally more proportion of variance. This is not surprising because these gene regions cover ∼0.45% of the genome and the SE of the estimate was ∼0.3% so that even if there is an enrichment of genetic variants in these gene regions, it is unable to be detected due to the lack of power. Larger sample size is required for such kind of analysis in the future.

In conclusion, we showed by whole genome estimation and partitioning analyses that, most human complex traits, if not all, appear to be highly polygenic, i.e. there are a large number of genetic variants segregating in the population with a small effect widely distributed across the whole genome. All the common SNPs on the Affymetrix 5.0 array explain approximately a third of heritability on average over all the 49 traits analysed in this study. The remaining unexplained two thirds of heritability could be due to causal variants including the common and rare ones that are not well tagged by SNPs on the array or possibly due to the heritability was over-estimated in the family/twin studies. The conclusion drawn from previous studies that heritability is not missing but due to many variants with small effects is not specific for human height in European populations but likely to be in common for most human complex traits and populations. Taken all together, it implies that although whole genome sequencing data will provide much denser genomic coverage than the current genotyping array and will therefore identify more associated variants and explain more genetic variance, large sample size is still essential.

## Materials and Methods

### The KARE cohort

This study used the data from the Korea Association Resource (KARE) project, which has been described elsewhere [15]. In brief, there were 10,038 individuals recruited from two community-based cohorts, 5,018 from Ansung and 5,020 from Ansan, in Gyeonggi Province, South Korea. The individuals were aged from 40 to 69 years old and born in 1931 to 1963. All the individuals were measured for a range of quantitative traits through epidemiological surveys, physical examinations and laboratory tests, including traits related to obesity, blood condition, pulse, bone mineral density, lipids, diabetes index, liver functions, lung functions and kidney functions. A description of the 49 traits used in this study is summarized in Table S1. We adjusted the phenotypes of each trait for age by simple regression and then standardized the residuals to *z*-scores, in each of the two cohorts (Ansung and Ansan) and in each gender group separately.

### Genotyped and imputed data

The genomic DNAs were isolated from peripheral blood drawn from the participants and were genotyped with 500,568 SNPs on the Affymetrix 5.0 genotyping array [15]. We excluded the SNPs with missingness rate >5%, minor allele frequency (MAF)<0.01, and Hardy-Weinberg equilibrium (HWE) test *P* value<10^−6^ using PLINK [18], and retained 326,262 autosomal SNPs for further analysis. The KARE GWAS data had been imputed to HapMap2 CHB and JPT panels [19]. After removing SNPs with MAF<0.01 and SNP missing rate >0.05, there were 2,153,258 genotyped/imputed SNPs [15].

### Estimating and partitioning genetic variance using SNP data

We estimated the genetic relationship matrix (GRM) between all pairs of individuals from all the genotyped SNPs and excluded one of each pair of individuals with estimated relationship >0.025 retaining 7,170 unrelated individuals. For each trait, we then estimated the variance that can be captured by all SNPs using the restricted maximum likelihood (REML) approach in mixed linear model 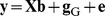, where **y** is a vector of phenotypes, **b** is a vector of fixed effects with its incidence matrix **X**, 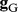 is a vector of aggregate effects of all SNPs, and 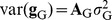 with **A**_G_ being the SNP-derived GRM and 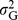 being the additive genetic variance. The proportion of variance explained by all SNPs is defined as 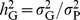 with 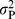 being the phenotypic variance. Details of the model and parameter estimation have been described elsewhere [10], [14]. In addition, using the same method as above but allowing to fit multiple genetic components simultaneously in the model, we partitioned 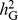 into the contributions of genic (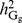) and intergenic (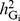) regions of the whole genome [11] and averaged the estimates of 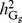 and 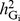 across all the traits. The genic regions were defined as ±0 kb, ±20 kb and ±50 kb of the 3′ and 5′ UTRs. A total of 135,491, 175,637 and 205,901 SNPs were located within the boundaries of 12,310, 15,140 and 15,274 protein-coding genes for the three definitions (±0 kb, ±20 kb and ±50 kb), respectively, which covered 36.1%, 49.2% and 58.9% of the genome.

## Supporting Information

Figure S1 Principal component analysis (PCA). The genotype data of the KARE cohort (8,842 individuals) was combined with the data from the HapMap3 project [20]. There are 1,397 individuals from 11 populations in the HapMap3 data. PCA was performed on the combined set of 10,239 individuals with ∼296K SNPs in common between KARE and HapMap3. Population codes shown in the figure are as follows: KOR-Korean in Ansan and Ansung, Korea; ASW-African ancestry from Southwest USA; CEU-Utah residents with Northern and Western European ancestry from the CEPH collection; CHB-Han Chinese in Beijing, China; CHD-Chinese in Metropolitan Denver, Colorado; GIH-Gujarati Indians in Houston, USA; JPT-Japanese in Tokyo, Japan; LWK-Luhya in Webuye, Kenya; MEX-Mexican ancestry in Los Angeles, USA; MKK-Massai in Kinyawa, Kenya; TSI-Tuscans, Italy; YRI-Yoruba in Ibadan, Nigeria. Plotted are eigenvector 1 against eigenvector 2 from PCA. The KARE cohort is overlapped with the three Eastern Asian samples in HapMap3 (CHB, CHD and JPT).
(PDF)

Figure S2 Pairwise phenotypic correlations between the 49 traits. The traits are classified into 10 groups: obesity, blood pressure & pulse, BMD, lipids, diabetes index, blood cell count, blood ions, liver function, lung function, and kidney function. The phenotypic correlations between traits in the same groups are stronger than those in different groups. From a principal component analysis of the phenotypic correlation matrix, the first 33 eigenvectors explain >95% of variance.
(PDF)

Figure S3 Variance explained by all SNPs estimated in the present study against the heritability estimates from pedigree analyses in literatures for the 49 traits. The regression slope is 0.137 (*P* = 0.017) and the regression *R* ^2^ is 0.131. Detailed information can be found in Table S1.
(PDF)

Figure S4 The observed proportion of SNPs with p-values passed a threshold p-value from genome-wide association analysis vs. the expected value (i.e. the threshold p-value). Shown on both axes are on the −log10 scale. A) −log10(*θ*_P_) value averaged across 47 traits (all traits except INS0 and HOMA) are plotted. B) −log10(*θ*_P_) of all the 47 traits are plotted.
(PDF)

Figure S5 Estimate of variance explained by each chromosome against chromosome length for each of the 47 traits (all traits except INS0 and HOMA).
(PDF)

Figure S6 Proportion of variance attributed to each chromosome averaged across traits against chromosome length when the number of SNPs on each chromosome is equal. There are 3500 SNPs randomly sampled from each chromosome. The estimate of variance explained by each chromosome is an average across all traits.
(PDF)

Figure S7 The estimates of variance explained by individual chromosomes against chromosome length for the 47 traits (all traits except INS0 and HOMA).
(PDF)

Figure S8 Manhattan plot of GWAS results for the traits with single variants of large effects. Panels A), B), C) and D) are for traits GLU60, HBA1C, RBC and RGTP, respectively.
(PDF)

Table S1 Summary description of the 49 traits in the KARE cohort.
(PDF)

Table S2 Estimates of variance explained by all SNPs for the 49 traits.
(PDF)

Table S3 Variance explained by all the SNPs on individual chromosomes for the 49 traits but HOMA and INS0.
(PDF)

Table S4 Estimates of the variance explained by all the genic and intergenic SNPs averaged across the 47 traits (all traits except INS0 and HOMA). A genic region is defined as ±0 kb, ±20 kb and ±50 kb of the 3′ and 5′ UTRs of a gene.
(PDF)

Table S5 Numbers of genic and intergenic SNPs on each chromosome.
(PDF)

Table S6 Estimate of heritability from a pedigree analysis for 11 traits. Data and analysis are described in Text S1.
(PDF)

Table S7 Estimates of variance explained by SNPs at the gene regions that are involved in insulin signaling pathway for 11 lipids and diabetes related traits.
(PDF)

Text S1 Difference between the estimates of variance explained by all SNPs in Europeans and in Koreans.
(PDF)

Text S2 Difference in number of SNPs and MAF distribution between genic and intergenic SNPs.
(PDF)
