# Supplementary figures and images for: Ubiquitous Polygenicity of Human Complex Traits: Genome-Wide Analysis of 49 Traits in Koreans
Source: PLoS Genet. 2013 Mar 7;9(3):e1003355. doi: 10.1371/journal.pgen.1003355 (PMC3591292; doi:10.1371/journal.pgen.1003355)

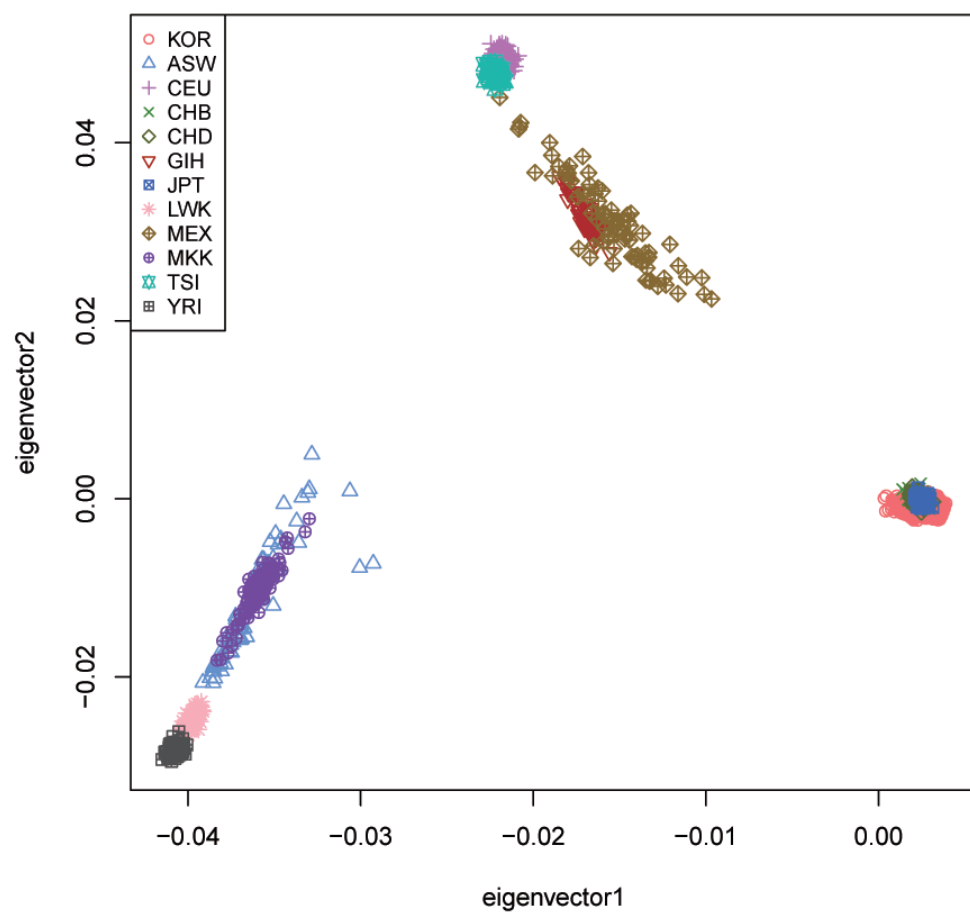

Supplement: Figure S1 — Principal component analysis (PCA). The genotype data of the KARE cohort (8,842 individuals) was combined with the data from the HapMap3 project [20]. There are 1,397 individuals from 11 populations in the HapMap3 data. PCA was performed on the combined set of 10,239 individuals with ∼296K SNPs in common between KARE and HapMap3. Population codes shown in the figure are as follows: KOR-Korean in Ansan and Ansung, Korea; ASW-African ancestry from Southwest USA; CEU-Utah residents with Northern and Western European ancestry from the CEPH collection; CHB-Han Chinese in Beijing, China; CHD-Chinese in Metropolitan Denver, Colorado; GIH-Gujarati Indians in Houston, USA; JPT-Japanese in Tokyo, Japan; LWK-Luhya in Webuye, Kenya; MEX-Mexican ancestry in Los Angeles, USA; MKK-Massai in Kinyawa, Kenya; TSI-Tuscans, Italy; YRI-Yoruba in Ibadan, Nigeria. Plotted are eigenvector 1 against eigenvector 2 from PCA. The KARE cohort is overlapped with the three Eastern Asian samples in HapMap3 (CHB, CHD and JPT). (PDF) [file pgen.1003355.s001.pdf]

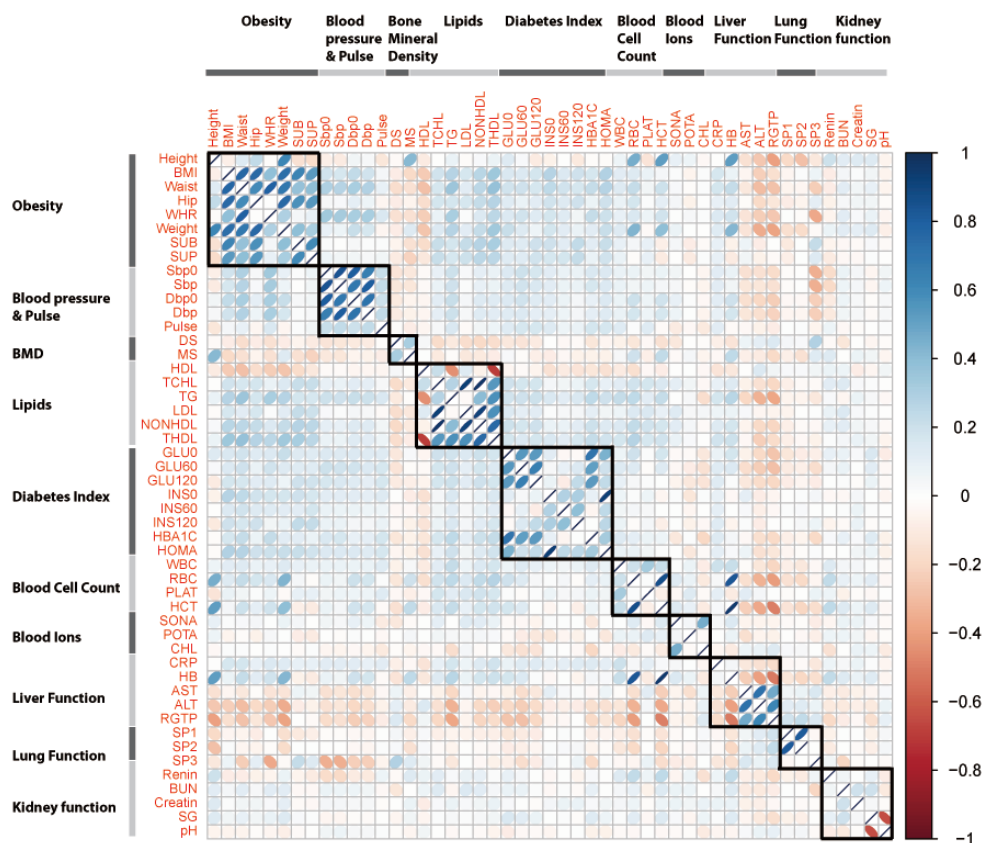

Supplement: Figure S2 — Pairwise phenotypic correlations between the 49 traits. The traits are classified into 10 groups: obesity, blood pressure & pulse, BMD, lipids, diabetes index, blood cell count, blood ions, liver function, lung function, and kidney function. The phenotypic correlations between traits in the same groups are stronger than those in different groups. From a principal component analysis of the phenotypic correlation matrix, the first 33 eigenvectors explain >95% of variance. (PDF) [file pgen.1003355.s002.pdf]

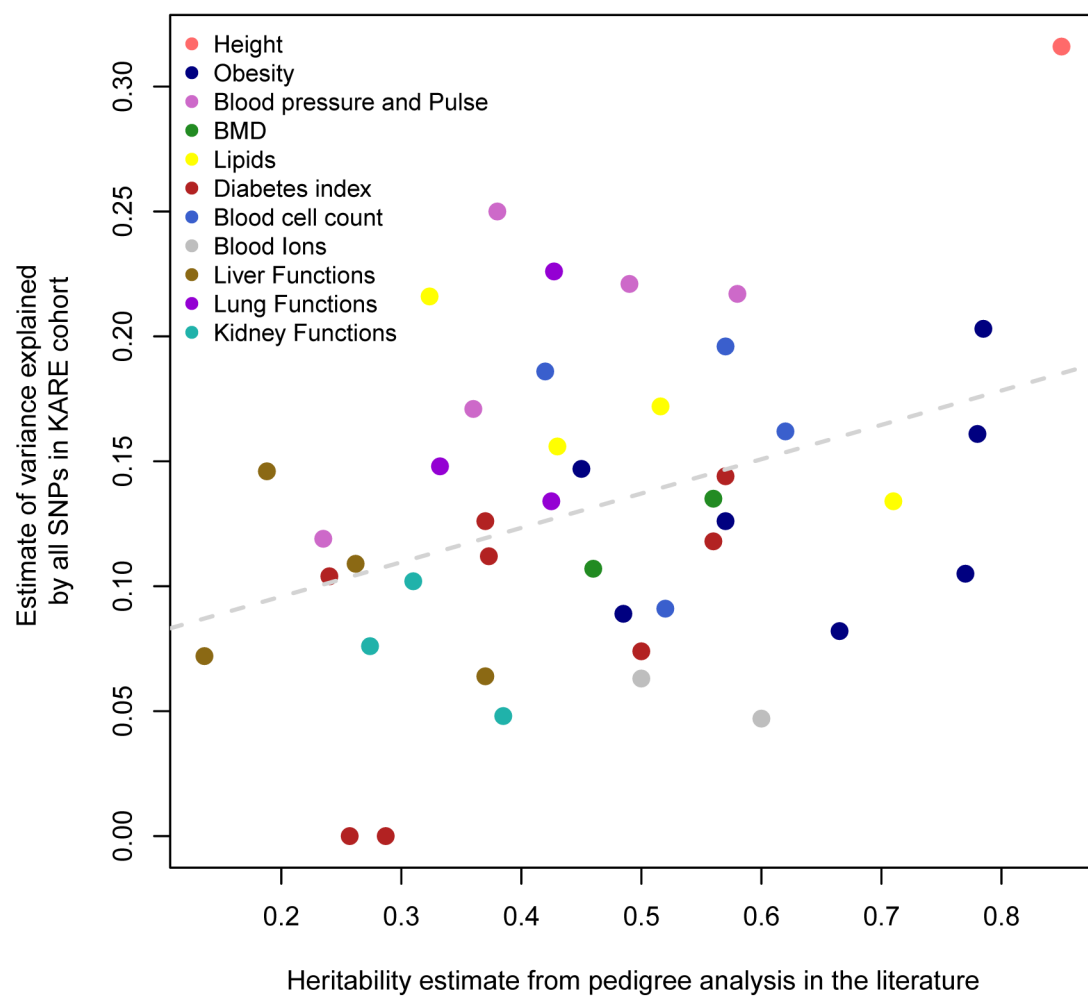

Supplement: Figure S3 — Variance explained by all SNPs estimated in the present study against the heritability estimates from pedigree analyses in literatures for the 49 traits. The regression slope is 0.137 (P = 0.017) and the regression R 2 is 0.131. Detailed information can be found in Table S1. (PDF) [file pgen.1003355.s003.pdf]

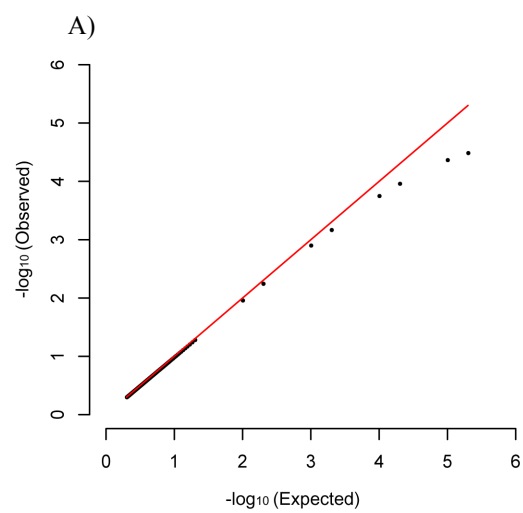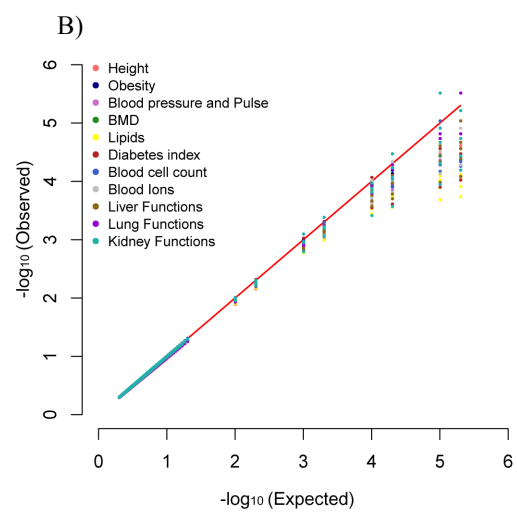

Supplement: Figure S4 — The observed proportion of SNPs with p-values passed a threshold p-value from genome-wide association analysis vs. the expected value (i.e. the threshold p-value). Shown on both axes are on the −log10 scale. A) −log10(θ P) value averaged across 47 traits (all traits except INS0 and HOMA) are plotted. B) −log10(θ P) of all the 47 traits are plotted. (PDF) [file pgen.1003355.s004.pdf]

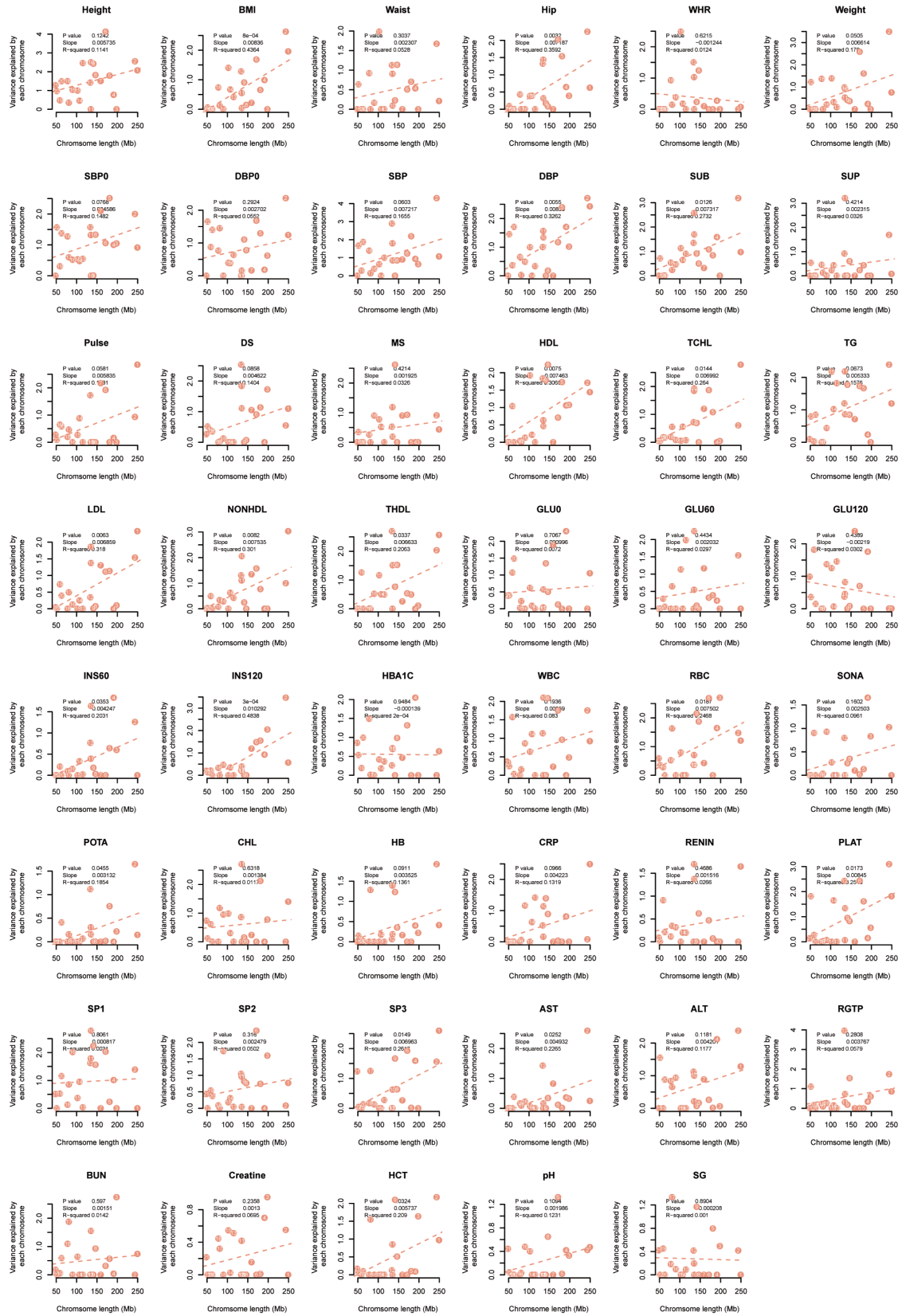

Supplement: Figure S5 — Estimate of variance explained by each chromosome against chromosome length for each of the 47 traits (all traits except INS0 and HOMA). (PDF) [file pgen.1003355.s005.pdf]

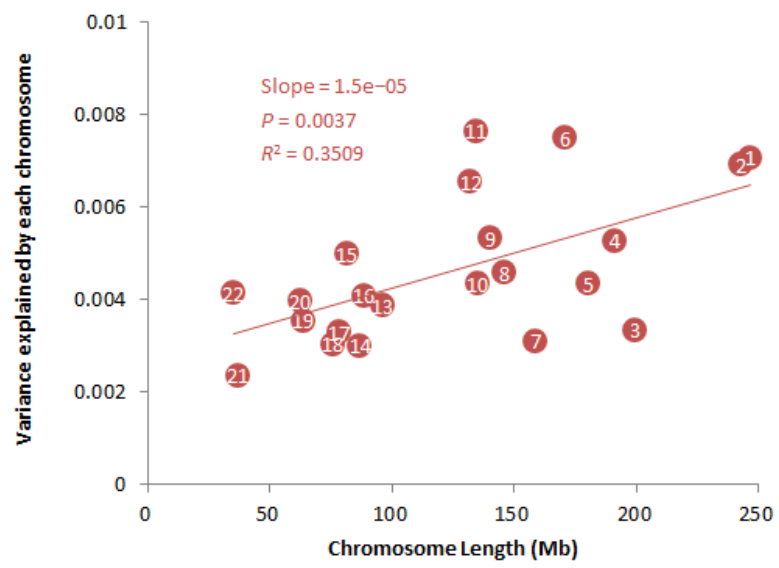

Supplement: Figure S6 — Proportion of variance attributed to each chromosome averaged across traits against chromosome length when the number of SNPs on each chromosome is equal. There are 3500 SNPs randomly sampled from each chromosome. The estimate of variance explained by each chromosome is an average across all traits. (PDF) [file pgen.1003355.s006.pdf]

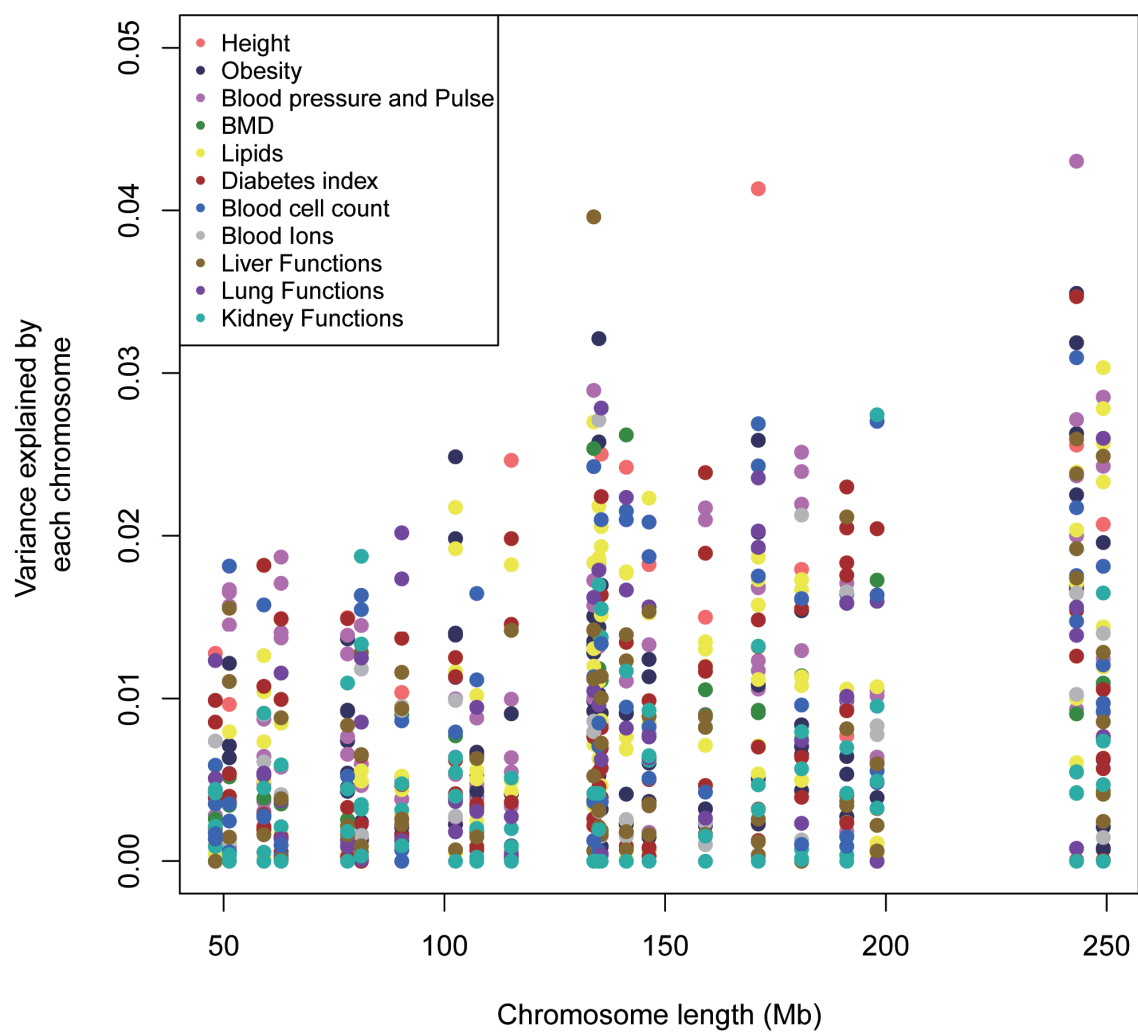

Supplement: Figure S7 — The estimates of variance explained by individual chromosomes against chromosome length for the 47 traits (all traits except INS0 and HOMA). (PDF) [file pgen.1003355.s007.pdf]

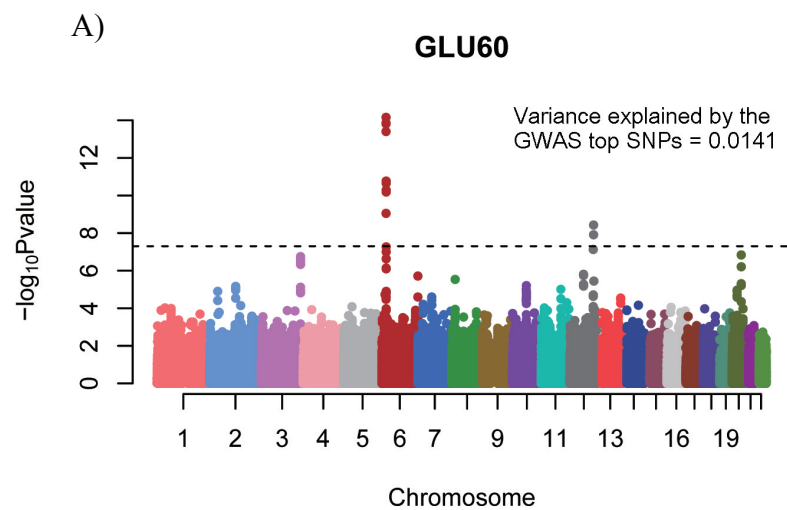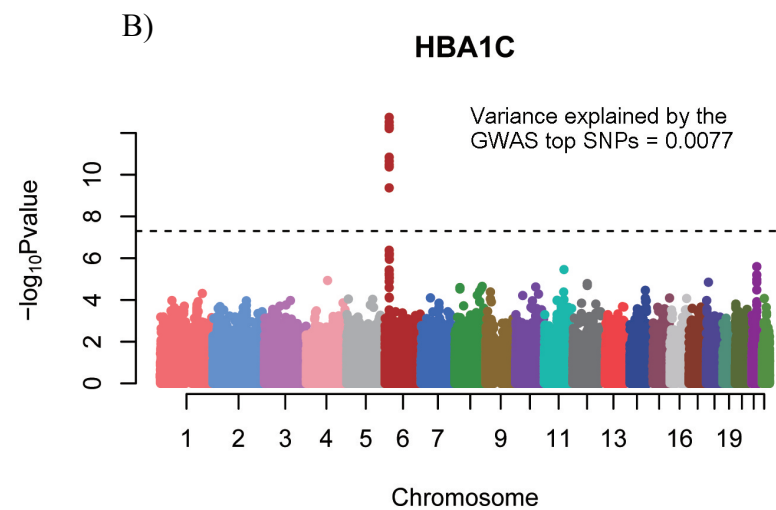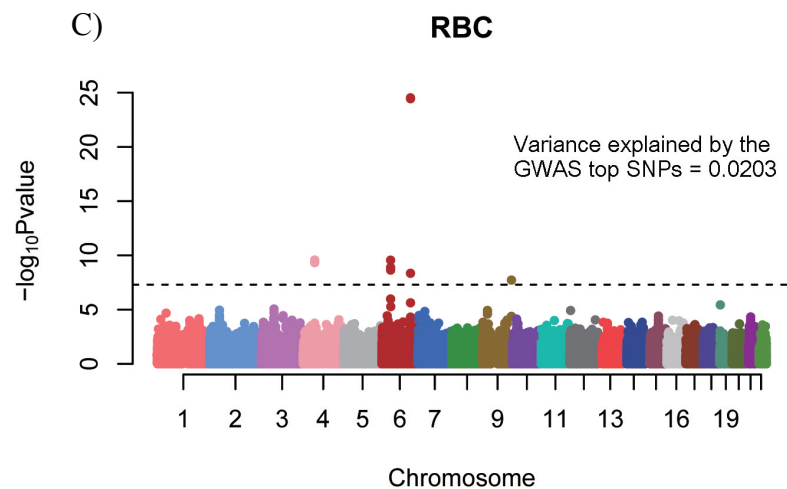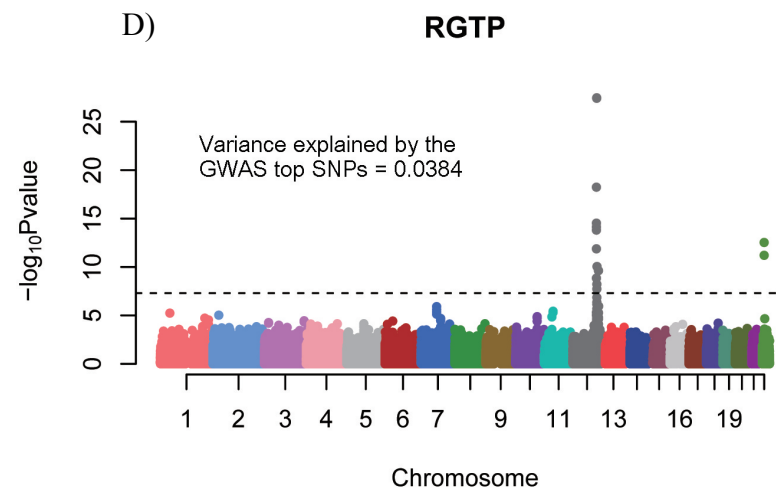

Supplement: Figure S8 — Manhattan plot of GWAS results for the traits with single variants of large effects. Panels A), B), C) and D) are for traits GLU60, HBA1C, RBC and RGTP, respectively. (PDF) [file pgen.1003355.s008.pdf]
