# Supplementary material for: Ubiquitous Polygenicity of Human Complex Traits: Genome-Wide Analysis of 49 Traits in Koreans
Source: PLoS Genet. 2013 Mar 7;9(3):e1003355. doi: 10.1371/journal.pgen.1003355 (PMC3591292; doi:10.1371/journal.pgen.1003355)
Supplement: Table S1 — Summary description of the 49 traits in the KARE cohort. (PDF) [file pgen.1003355.s009.pdf]

Group	Trait	Detailed Description
**Obesity**	Height	Height is a classical polygenic trait, measured in cm scale [1].
	BMI	BMI, weight in kilograms divided by height in meters squared, is a widely used index of obesity. Obesity itself is one of the major risk factors for common complex diseases such as T2DM, heart disease, metabolic syndrome, hypertension, stroke, and some forms of cancer [1].
	Waist	Waist was measured in cm scale.
	Hip	Hip circumference was measured in cm scale.
	WHR	WHR is the ratio of the waist to hip circumferences and is a commonly used measure of the central accumulation of fat [1].
	Weight	Weight was measured in kg scale.
	SUB<br>SUP	Subscapular and suprailiac skinfold thickness values are measurements for upper central body fat distribution around the waist. It is measured just below the angle of the left scapula with the fold either in a vertical line or slightly inclined [2]. The suprailiac measure is a diagonal fold taken midway between the hip joint and the bottom of the rib cage. Natural logarithmic transformation and square root transformation were applied to the subscapular and suprailiac measures respectively [3].
**Blood Pressure**	SBP0<br>SBP<br>DBP0<br>DBP	Blood pressure refers to the force exerted by circulating blood on the walls of blood vessels and constitutes one of the principal vital signs. Typical values for a resting, healthy adult human are approximately 120 mmHg systolic and 80 mmHg diastolic although there is substantial individual variation. In this study, diastolic and systolic BP values analyzed were the averages of three recumbent measurements. Genetic factors are thought to play a prominent role in the development of essential hypertension, though few, if any, robust associations have been found [1]. Both diastolic and systolic BP values were measured in sitting (SBP0, DBP0) and lying positions (SBP, DBP).
	Pulse	Pulse rate, also called heart rate, is the frequency of the heart beat (beats per minute, BPM) [1]. A normal pulse rate for a healthy adult, while resting, ranges from 60 to 80 BPM [4]. Changes in pulse rate may be caused by heart disease or problems in heart [1].
**BMD**	DS<br>MS	Bone density is a proxy measurement for bone strength, which is the resistance to fracture, widely used to screen for osteoporosis. Bone density was estimated by T-score by dividing the difference of measured SOS (speed of sound) from mean SOS in healthy young adult population by the standard deviation of SOS in young adult population. Bone SOS was measured by quantitative ultrasound at distal radius or mid-shaft tibia in the subjects of the KARE GWAS [1].
**Lipids**	HDL<br>TCHL<br>TG<br>LDL<br>NONHDL<br>THDL	Abbreviations are as follows: HDL, high-density lipoprotein cholesterol; TCHL, total cholesterol; TG, triglyceride; LDL, low-density lipoprotein cholesterol. TCHL, HDL, and TG were extracted from human blood. LDL was calculated by Frederewald formula when TG level was under 400 mg/dl. Otherwise, LDL was considered to be missing, because it could not be calculated from formula [5]. The Fredewald formula is LDL-C=TC-HDL-C-(TG/5) [6]. Before modeling, HDL and TG traits were natural log transformed to approximate a normal distribution. NONHDL is HDL subtracted from TG. Finally, TDHL is the natural logarithmic transformed measure of TG divided by HDL.
**Diabetes Index**	GLU0<br>GLU60<br>GLU120<br>INS0<br>INS60<br>INS120<br>HBA1C<br>HOMA	Type 2 diabetes (T2D) is a state of chronic hyperglycemia defined as elevated glucose levels measured either when fasting or 2 h after glucose challenge during oral glucose tolerance test (OGTT) [7]. Subject underwent a 75-g OGTT with blood sampling at fasting (GLU0, INS0), and 60 (GLU60, INS60) and 120 min (GLU120, INS120) after the oral glucose load to measure the glucose and insulin levels [8]. All of the blood glucose and insulin levels were natural log transformed to approximate a normal distribution. Homoeostasis model assessment for insulin resistance (HOMA-IR) was assessed using the formula: HOMA-IR=fasting serum insulin (mU/ml) × fasting glucose (mmol/l)/22.5. [9] The HOMA-IR was square root transformed to approximate a normal distribution. HBA1C, glycosylated hemoglobin, reflects blood glucose concentrations over the preceding 8 to 12 weeks [10].
**Blood Cell Count**	WBC<br>RBC<br>PLAT<br>HCT	The hematopoietic system has various functions including oxygen transport, immunity, blood wall surveillance, homeostasis, and wound curing. Hematological parameters include the number of white blood cells (WBC; 10^3/μL), red blood cells (RBC; 10^3/μL), platelets (PLAT; 10^3/μL), and hematocrit (HCT; %) [11] from the whole blood. The number of WBC was natural log transformed to approximate a normal distribution.
**Blood Ions**	SONA<br>POTA<br>CHL	Abbreviations are as follows: SONA, sodium; potassium; CHL, chloride. Most of the sodium in the body (about 85%) is found in blood and lymph fluid. Sodium is both an electrolyte and mineral. It helps keep the water and electrolyte balance of the body. Other electrolytes, such as potassium and chloride, were checked in the subjects at the same time as a blood test for sodium. The level of potassium is inversely related to the sodium level. Potassium is also both

		an electrolyte and a mineral. Sodium and potassium levels are known to affect the risk of hypertension[12]. Chloride is one of the most important electrolytes in the blood.
**Liver Functions**	CRP<br>HB<br>AST<br>ALT<br>RGTP	C-reactive protein (CRP) is a plasma protein synthesized by the liver and found in the blood, and thus a sensitive and dynamic systemic marker of inflammation [13]. The distribution of CRP concentrations (mg/L) was positively skewed and hence CRP concentration was natural log transformed for all analyses. Hemoglobin (HB) was measured in g/dl scale and extracted from whole blood. Alanine transaminase (ALT) is a crucial enzyme found predominately in the liver. Increased activity of ALT in serum suggests hepatocyte damage [14]. When a cell is damaged, it leaks this enzyme into the blood, where it is measured. Aspartate transaminase (AST) is similar to ALT in that it is another enzyme associated with liver parenchymal cells. It is a less specific indicator of liver inflammation than the AST, as AST level change can be caused by diseases affecting other organs. The value of ALT was transformed to 1/square root (y) while AST was transformed to 1/(y). Gamma-glutamyltranspeptidase (RGTP) is a membrane-bound glycoprotein that transfers gamma-glutamyl functional groups between peptides or amino acids [15]. It is found in many tissues, but the most notable one being the liver, and has significance in medicine as a diagnostic marker [16]. The value of RGTP was transformed to 1/square root (y).
**Lung Functions**	SP1<br>SP2<br>SP3	Forced vital capacity (FVC) is the volume of air that can forcibly be blown out after full inspiration, measured in liters. FVC is the most basic maneuver in spirometry tests, and FEV1 is the forced expiratory volume in one second [17]. All values were corrected and expressed as a percentage of those predicted (%pred) for age, sex and height. The predicted values for FVC (FVC_%PRED), FEV1 (FEV1_%PRED), and FEV1/FVC (FEV1/FVC_PRED) from the cohorts were used, and those values are abbreviated as SP1, SP2, SP3, respectively.
**Kidney Functions**	RENIN<br>Bun<br>Creatine<br>SG<br>pH	Abbreviations are as follows: BUN, blood urea nitrogen; SG, specific gravity of urine. Plasma renin activity (PRA) is a measure of the activity of the plasma enzyme renin which plays a crucial role in the body's regulation of blood pressure and urine output [18]. The PRA was natural log transformed to approximate a normal distribution. The level of blood urea nitrogen (BUN) and creatine show how well kidneys are working. The plasma levels of the BUN and creatine were measured in serum, using mg/dL and the BUN was log transformed before all analyses.<br>Specific gravity of urine (SG) and pH of urine measure the kidney's ability to concentrate or dilute urine in relation to plasma [19,20] .

References

1. Cho YS, Go MJ, Kim YJ, Heo JY, Oh JH, et al. (2009) A large-scale genome-wide association study of Asian populations uncovers genetic factors influencing eight quantitative traits. Nature genetics 41: 527-534.
2. Tanner JM, Whitehouse RH (1975) Revised standards for triceps and subscapular skinfolds in British children. Archives of Disease in Childhood 50: 142-145.
3. Kim J, Namkung J, Lee S, Park T (2010) Application of Structural Equation Models to Genome-wide Association Analysis. Genomics & Informatics 8: 150-158.
4. Cook S, Togni M, Schaub MC, Wenaweser P, Hess OM (2006) High heart rate: a cardiovascular risk factor? European heart journal 27: 2387-2393.
5. Huh IS, Oh S, Lee E, Park T. Compairing quantitative trait analysis to qualitative trait analysis for complex traits disease: A genome wide association study for hyperlipidemia; 2010. IEEE. pp. 341-346.
6. Friedewa.Wt, Fredrick.Ds, Levy RI (1972) Estimation of Concentration of Low-Density Lipoprotein Cholesterol in Plasma, without Use of Preparative Ultracentrifuge. Clinical Chemistry 18: 499-&.
7. Saxena R, Hivert MF, Langenberg C, Tanaka T, Pankow JS, et al. (2010) Genetic variation in GIPR influences the glucose and insulin responses to an oral glucose challenge. Nature genetics 42: 142-148.
8. Wu KD, Hsiao CF, Ho LT, Sheu W, Pei D, et al. (2002) Clustering and heritability of insulin resistance in

Chinese and Japanese hypertensive families: a Stanford-Asian Pacific Program in Hypertension and Insulin Resistance sibling study. Hypertension research: official journal of the Japanese Society of Hypertension 25: 529.

9. Matthews DR, Hosker JP, Rudenski AS, Naylor BA, Treacher DF, et al. (1985) Homeostasis Model Assessment - Insulin Resistance and Beta-Cell Function from Fasting Plasma-Glucose and Insulin Concentrations in Man. Diabetologia 28: 412-419.
10. Little RR, Sacks DB (2009) HbA1c: how do we measure it and what does it mean? Current Opinion in Endocrinology, Diabetes and Obesity 16: 113.
11. Kim KK, Cho YS, Cho NH, Shin C, Kim JW (2010) A Genome-wide Association Study of Copy Number Variation in Hematological Parameters in the Korean Population. Genomics & Informatics 8: 122-130.
12. Sacks FM, Svetkey LP, Vollmer WM, Appel LJ, Bray GA, et al. (2001) Effects on blood pressure of reduced dietary sodium and the Dietary Approaches to Stop Hypertension (DASH) diet. New England Journal of Medicine 344: 3-10.
13. Kaptoge S, Di Angelantonio E, Lowe G, Pepys MB, Thompson SG, et al. (2010) C-reactive protein concentration and risk of coronary heart disease, stroke, and mortality: an individual participant meta-analysis. Lancet 375: 132.
14. Hamad EM, Taha SH, Dawood AGIA, Sitohy MZ, Abdel-Hamid M (2011) Protective effect of whey proteins against nonalcoholic fatty liver in rats. Lipids in health and disease 10: 57.
15. Sahm DF, Murray JL, Munson PL, Nordquist RE, Lerner MP (1983) Gamma−glutamyltranspeptidase levels as an aid in the management of human cancer. Cancer 52: 1673-1678.
16. Dragosics B, Ferenci P, Pesendorfer F, Wewalka F (1976) Gamma-glutamyltranspeptidase (GGTP): its relationship to other enzymes for diagnosis of liver disease. Progress in liver diseases 5: 436.
17. Knudson RJ, Lebowitz M, Holberg C, Burrows B (1983) Changes in the normal maximal expiratory flow-volume curve with growth and aging. The American review of respiratory disease 127: 725.
18. Tuck ML, Sowers J, Dornfeld L, Kledzik G, Maxwell M (1981) The effect of weight reduction on blood pressure, plasma renin activity, and plasma aldosterone levels in obese patients. New England Journal of Medicine 304: 930-933.
19. Burkhardt AE, Johnston KG, Waszak CE, Jackson CE, Shafer SR (1982) A reagent strip for measuring the specific gravity of urine. Clinical Chemistry 28: 2068-2072.
20. Askergren A, Allgén LG, Karlsson C, Lundberg I, Nyberg E (1981) Studies on kidney function in subjects exposed to organic solvents. Acta Medica Scandinavica 209: 479-483.
