# Supplementary material for: Ubiquitous Polygenicity of Human Complex Traits: Genome-Wide Analysis of 49 Traits in Koreans
Source: PLoS Genet. 2013 Mar 7;9(3):e1003355. doi: 10.1371/journal.pgen.1003355 (PMC3591292; doi:10.1371/journal.pgen.1003355)
Supplement: Table S2 — Estimates of variance explained by all SNPs for the 49 traits. (PDF) [file pgen.1003355.s010.pdf]

Group	Trait	*n*	Genotyped data		Imputed data		[c] h^2_{Gsig}	[d] Heritability
			[a] h^2_G (SE)	*P*	[b] h^2_G (SE)	*P*		
	Height	717	0.316 (0.042)	2.1e-15	0.283 (0.040)	1.0E-13	-	0.8~0.9[1]
**Obesity**	BMI	716	0.147 (0.041)	1.1e-04	0.132 (0.039)	2.0E-04	-	0.25-0.40, 0.70[2]
	Waist	716	0.105 (0.040)	4.1e-03	0.098 (0.039)	5.0E-03	-	0.72~0.82[3],0.77[4]
	Hip	716	0.126 (0.040)	7.0e-04	0.157 (0.039)	1.0E-05	-	0.45~0.69[5]
	WHR	716	0.082 (0.040)	2.0e-02	0.131 (0.038)	1.0E-04	-	0.36~0.61[3]
	Weight	716	0.161 (0.040)	1.8e-05	0.152 (0.038)	1.0E-05	-	0.78[6]
	SUB	713	0.203 (0.041)	1.0e-07	0.232 (0.039)	2.0E-10	-	0.40~0.77[7]
	SUP	657	0.089 (0.043)	1.7e-02	0.169 (0.041)	4.0E-06	-	0.48, 0.49[8]
**Blood Pressure**	SBP0	717	0.221 (0.041)	1.1e-08	0.198 (0.039)	6.0E-08	0.005	0.34[9], 0.64[10]
	SBP	716	0.250 (0.041)	5.8e-11	0.227 (0.039)	7.0E-10	0.009	0.38[11]
	DBP0	716	0.217 (0.041)	3.7e-08	0.154 (0.039)	2.0E-05	0.005	0.29[9], 0.45~0.58[10]
	DBP	717	0.171 (0.041)	6.7e-06	0.189 (0.040)	8.0E-07	0.005	0.36[11]
	Pulse	716	0.119 (0.041)	1.6e-03	0.092 (0.039)	8.0E-03		0.13[9], 0.19,0.22,0.40[12]
**BMD**	DS	675	0.135 (0.043)	6.0e-04	0.157 (0.042)	7.0E-05	0.012	0.61[13], 0.51[14]
	MS	677	0.107 (0.042)	4.3e-03	0.099 (0.040)	6.0E-03	0.005	0.46[14]
**Lipids**	HDL	716	0.172 (0.041)	8.5e-06	0.190 (0.040)	5.0E-07	0.037	0.328[15],0.56[16], 0.66[17]
	TCHL	716	0.156 (0.040)	2.3e-05	0.196 (0.039)	4.0E-08	0.020	0.42[18], 0.44[15]
	TG	716	0.216 (0.041)	1.5e-08	0.236 (0.039)	9.0E-11	0.030	0.117[15],0.53[17]
	LDL	696	0.134 (0.041)	3.8e-04	0.178 (0.040)	1.0E-06	0.012	0.88[17], 0.54[19]
	NONHD	716	0.157 (0.040)	1.9e-05	0.191 (0.039)	6.0E-08	0.025	
	THDL	716	0.162 (0.040)	1.4e-05	0.173 (0.039)	1.0E-06	0.025	
**Diabetes**	GLU0	700	0.112 (0.041)	2.9e-03	0.134 (0.040)	3.0E-04	-	0.26[6], 0.28[20], 0.58[21]
	GLU60	682	0.104 (0.043)	7.2e-03	0.100 (0.041)	6.0E-03	0.014	0.24[22]
	GLU120	683	0.118 (0.042)	1.5e-03	0.102 (0.040)	4.0E-03	-	0.52[6], 0.60[21]
	INS0	700	0.000 (0.040)	5.0e-01	0.000 (0.038)	5.0E-01	-	0.26[6], 0.08[20], 0.43[21]
	INS60	682	0.074 (0.042)	3.9e-02	0.104 (0.040)	4.0E-03	-	0.50[6]
	INS120	682	0.144 (0.043)	3.8e-04	0.234 (0.041)	2.0E-10	0.005	0.57[21]
	HBA1C	716	0.126 (0.040)	5.8e-04	0.124 (0.039)	5.0E-04	0.008	0.37[23]
	HOMA	700	0.000 (0.040)	5.0e-01	0.000 (0.038)	5.0E-01	-	0.29~0.31[22],0.48[24],
**Blood Cell Count**	WBC	716	0.162 (0.041)	2.3e-05	0.155 (0.039)	3.0E-05	0.005	0.62[23]
	RBC	716	0.186 (0.041)	1.1e-06	0.173 (0.039)	2.0E-06	0.020	0.42[23]
	PLAT	716	0.196 (0.041)	3.5e-07	0.216 (0.039)	5.0E-09	0.009	0.57[23]
	HCT	716	0.091 (0.040)	9.6e-03	0.076 (0.038)	2.0E-02	-	0.4~0.64[25-27]
**Blood Ions**	SONA	716	0.063 (0.039)	4.7e-02	0.075 (0.038)	2.0E-02	0.016	0.5[28]
	POTA	716	0.047 (0.039)	1.2e-01	0.057 (0.038)	7.0E-02	-	0.6[28]
	CHL	716	0.113 (0.039)	9.1e-04	0.136 (0.038)	8.0E-05	-	
**Liver Functions**	CRP	716	0.109 (0.039)	1.1e-03	0.131 (0.038)	9.0E-05	0.011	0.06[15], 0.28[29],0.45[30]
	HB	716	0.064 (0.039)	4.9e-02	0.050 (0.037)	8.0E-02	-	0.37[23]
	AST	716	0.072 (0.040)	3.0e-02	0.073 (0.038)	2.0E-02	0.005	0.14[15]
	ALT	716	0.146 (0.040)	7.4e-05	0.129 (0.038)	2.0E-04	-	0.19[15]
	RGTP	716	0.109 (0.040)	2.9e-03	0.089 (0.039)	1.0E-02	0.038	
**Lung Functions**	SP1	700	0.226 (0.043)	2.1e-08	0.251 (0.041)	7.0E-11	-	0.39[31],0.54[32],
	SP2	700	0.134 (0.041)	4.2e-04	0.166 (0.040)	6.0E-06	-	0.46[31],
	SP3	701	0.148 (0.041)	1.0e-04	0.132 (0.040)	3.0E-04	-	0.45[32],0.46,0.16[33],0.26[
**Kidney Functions**	RENIN	716	0.076 (0.039)	2.3e-02	0.110 (0.038)	1.0E-03	0.018	0.27[35]
	Bun	716	0.102 (0.040)	4.7e-03	0.092 (0.038)	7.0E-03	0.013	0.31[11]
	Creatine	716	0.048 (0.040)	1.1e-01	0.143 (0.037)	9.0E-06	0.017	0.24~0.53[36]
	SG	714	0.034 (0.039)	1.9e-01	0.047 (0.038)	1.0E-01	-	
	pH	714	0.039 (0.040)	1.7e-01	0.048 (0.038)	1.0E-01	-	

[a] Estimate of h^2_G (SE) using genotyped data; [b] Estimate of h^2_G (SE) using imputed data (data were imputed to HapMap2 CHB and JPT panels); [c] Estimate of variance explained by the top SNPs at P < 5e-8 in KARE GWAS. [b] Estimate of h^2 from pedigree analysis in literatures.

References

1. Visscher PM, Hill WG, Wray NR (2008) Heritability in the genomics era - concepts and misconceptions. Nature Reviews Genetics 9: 255-266.
2. Schousboe K, Willemsen G, Kyvik KO, Mortensen J, Boomsma DI, et al. (2003) Sex differences in heritability of BMI: a comparative study of results from twin studies in eight countries. Twin Research 6: 409-421.
3. Rose KM, Newman B, Mayer-Davis E, Selby JV (1998) Genetic and behavioral determinants of waist-hip ratio and waist circumference in women twins. Obesity research 6: 383.
4. Wardle J, Carnell S, Haworth C, Plomin R (2008) Evidence for a strong genetic influence on childhood adiposity despite the force of the obesogenic environment. The American journal of clinical nutrition 87: 398-404.
5. Jian WX, Long JR, Deng HW (2004) High heritability of bone size at the hip and spine in Chinese. Journal of human genetics 49: 87-91.
6. Poulsen P, Ohm Kyvik K, Vaag A, Beck-Nielsen H (1999) Heritability of type II (non-insulin-dependent) diabetes mellitus and abnormal glucose tolerance–a population-based twin study. Diabetologia 42: 139-145.
7. Selby J, Newman B, Quesenberry Jr C, Fabsitz R, King M, et al. (1989) Evidence of genetic influence on central body fat in middle-aged twins. Human biology; an international record of research 61: 179.
8. Schousboe K, Visscher P, Erbas B, Kyvik KO, Hopper J, et al. (2003) Twin study of genetic and environmental influences on adult body size, shape, and composition. International Journal of Obesity 28: 39-48.
9. Adeyemo AA, Omotade OO, Rotimi CN, Luke AH, Tayo BO, et al. (2002) Heritability of blood pressure in Nigerian families. Journal of hypertension 20: 859.
10. Snieder H, Harshfield GA, Treiber FA (2003) Heritability of blood pressure and hemodynamics in African- and European-American youth. Hypertension 41: 1196.
11. Chen YC, Guo X, Raffel LJ, Xiang AH, Fang B, et al. (2008) Carotid intima-media thickness (cIMT) cosegregates with blood pressure and renal function in hypertensive Hispanic families. Atherosclerosis 198: 160-165.
12. Hsu FC, Zaccaro DJ, Lange LA, Arnett DK, Langefeld CD, et al. (2005) The impact of pedigree structure on heritability estimates for pulse pressure in three studies. Human heredity 60: 63-72.
13. Arden N, Baker J, Hogg C, Baan K, Spector T (1996) The heritability of bone mineral density, ultrasound of the calcaneus and hip axis length: a study of postmenopausal twins. Journal of Bone and Mineral Research 11: 530-534.
14. Wang X, Wheeler VW, Patrick AL, Bunker CH, Zmuda JM (2007) Genetic and Environmental Determinants of Volumetric and Areal BMD in Multi−Generational Families of African Ancestry: The Tobago Family Health Study. Journal of Bone and Mineral Research 22: 527-536.
15. Herbeth B, Samara A, Ndiaye C, Marteau JB, Berrahmoune H, et al. (2010) Metabolic syndrome-related

composite factors over 5years in the STANISLAS Family Study: Genetic heritability and common environmental influences. Clinica Chimica Acta 411: 833-839.

16. Cohen J, Wang Z, Grundy S, Stoesz M, Guerra R (1994) Variation at the hepatic lipase and apolipoprotein AI/CIII/AIV loci is a major cause of genetically determined variation in plasma HDL cholesterol levels. Journal of Clinical Investigation 94: 2377.
17. AUSTIN MA, KING MC, BAWOL RD, HULLEY SB, FRIEDMAN GD (1987) Risk factors for coronary heart disease in adult female twins. American journal of epidemiology 125: 308.
18. HUNT SC, HASSTEDT SJ, KUIDA H, STULTS BM, HOPKINS PN, et al. (1989) Genetic heritability and common environmental components of resting and stressed blood pressures, lipids, and body mass index in Utah pedigrees and twins. American journal of epidemiology 129: 625.
19. Austin MA, Newman B, Selby JV, Edwards K, Mayer EJ, et al. (1993) Genetics of LDL subclass phenotypes in women twins. Concordance, heritability, and commingling analysis. Arteriosclerosis, thrombosis, and vascular biology 13: 687-695.
20. Henkin L, Bergman RN, Bowden DW, Ellsworth DL, Haffner SM, et al. (2003) Genetic Epidemiology of Insulin Resistance and Visceral Adiposity* 1:: The IRAS Family Study Design and Methods. Annals of epidemiology 13: 211-217.
21. Wu KD, Hsiao CF, Ho LT, Sheu W, Pei D, et al. (2002) Clustering and heritability of insulin resistance in Chinese and Japanese hypertensive families: a Stanford-Asian Pacific Program in Hypertension and Insulin Resistance sibling study. Hypertension research: official journal of the Japanese Society of Hypertension 25: 529.
22. Mills G, Avery P, McCarthy M, Hattersley A, Levy J, et al. (2004) Heritability estimates for beta cell function and features of the insulin resistance syndrome in UK families with an increased susceptibility to type 2 diabetes. Diabetologia 47: 732-738.
23. Garner C, Tatu T, Reittie J, Littlewood T, Darley J, et al. (2000) Genetic influences on F cells and other hematologic variables: a twin heritability study. Blood 95: 342.
24. Bayoumi RA, Al-Yahyaee SAS, Albarwani SA, Rizvi SG, Al-Hadabi S, et al. (2007) Heritability of Determinants of the Metabolic Syndrome among Healthy Arabs of the Oman Family Study&ast. Obesity 15: 551-556.
25. Whitfield J, Martin N, Rao D (1985) Genetic and environmental influences on the size and number of cells in the blood. Genetic epidemiology 2: 133-144.
26. Evans DM, Frazer IH, Martin NG (1999) Genetic and environmental causes of variation in basal levels of blood cells. Twin Research 2: 250-257.
27. Garner C, Mitchell J, Hatzis T, Reittie J, Farrall M, et al. (1998) Haplotype mapping of a major quantitative-trait locus for fetal hemoglobin production, on chromosome 6q23. The American Journal of Human Genetics 62: 1468-1474.
28. Whitfield J, Martin N, Rao D (1985) Genetic and environmental causes of variation in renal tubular handling of sodium and potassium: a twin study. Genetic epidemiology 2: 17-27.
29. Rhodes B, Meek J, Whittaker J, Vyse T (2008) Quantification of the Genetic Component of Basal C–

Reactive Protein Expression in SLE Nuclear Families. Annals of human genetics 72: 611-620.

30. Fox ER, Benjamin EJ, Sarpong DF, Rotimi CN, Wilson JG, et al. (2008) Epidemiology, heritability, and genetic linkage of C-reactive protein in African Americans (from the Jackson Heart Study). The American journal of cardiology 102: 835-841.
31. Palmer L, Knuiman M, Divitini M, Burton P, James A, et al. (2001) Familial aggregation and heritability of adult lung function: results from the Busselton Health Study. European Respiratory Journal 17: 696-702.
32. Wilk JB, Djousse L, Arnett DK, Rich SS, Province MA, et al. (2000) Evidence for major genes influencing pulmonary function in the NHLBI family heart study. Genetic epidemiology 19: 81-94.
33. Hukkinen M, Kaprio J, Broms U, Viljanen A, Kotz D, et al. (2011) Heritability of Lung Function: A Twin Study Among Never-Smoking Elderly Women. Twin Research and Human Genetics 14: 401-407.
34. DeMeo D, Campbell E, Brantly M, Barker A, Eden E, et al. (2009) Heritability of lung function in severe alpha-1 antitrypsin deficiency. Hum Hered 67: 38-45.
35. Alvarez-Madrazo S, Padmanabhan S, Mayosi BM, Watkins H, Avery P, et al. (2009) Familial and phenotypic associations of the aldosterone renin ratio. Journal of Clinical Endocrinology & Metabolism 94: 4324.
36. Pattaro C, Aulchenko YS, Isaacs A, Vitart V, Hayward C, et al. (2009) Genome-wide linkage analysis of serum creatinine in three isolated European populations. Kidney international 76: 297-306.
