# Supplementary material for: Ubiquitous Polygenicity of Human Complex Traits: Genome-Wide Analysis of 49 Traits in Koreans
Source: PLoS Genet. 2013 Mar 7;9(3):e1003355. doi: 10.1371/journal.pgen.1003355 (PMC3591292; doi:10.1371/journal.pgen.1003355)
Supplement: Table S3 — Variance explained by all the SNPs on individual chromosomes for the 49 traits but HOMA and INS0. (PDF) [file pgen.1003355.s011.pdf]

Chr.		1	2	3	4	5	6	7	8	9	10	11	12	13	14	15	16	17	18	19	20	21	22	Total
L_C (Mb)		249.3	243.2	198.0	191.2	180.9	171.1	159.1	146.4	141.2	135.5	135.0	133.9	115.2	107.3	102.5	90.4	81.2	78.1	59.1	63.0	48.1	51.3	2881.0
Height	h^2_C	0.021	0.026	0.000	0.008	0.018	0.041	0.015	0.018	0.024	0.025	0.000	0.014	0.025	0.005	0.010	0.010	0.004	0.015	0.005	0.015	0.013	0.010	0.32
	SE	0.012	0.012	0.011	0.010	0.011	0.011	0.010	0.010	0.010	0.010	0.008	0.010	0.009	0.008	0.008	0.008	0.007	0.008	0.006	0.008	0.006	0.006	
BMI	h^2_C	0.020	0.026	0.000	0.010	0.007	0.017	0.002	0.002	0.009	0.000	0.006	0.013	0.004	0.007	0.014	0.000	0.001	0.007	0.000	0.000	0.000	0.001	0.14
	SE	0.012	0.013	0.010	0.010	0.010	0.010	0.009	0.009	0.009	0.010	0.009	0.010	0.008	0.008	0.009	0.008	0.007	0.008	0.006	0.007	0.006	0.005	
Waist	h^2_C	0.002	0.017	0.000	0.005	0.007	0.005	0.000	0.011	0.002	0.001	0.009	0.011	0.000	0.000	0.020	0.002	0.000	0.009	0.000	0.000	0.000	0.006	0.11
	SE	0.011	0.012	0.011	0.010	0.010	0.010	0.009	0.009	0.009	0.010	0.009	0.009	0.008	0.007	0.009	0.008	0.007	0.008	0.006	0.007	0.005	0.006	
Hip	h^2_C	0.006	0.023	0.004	0.006	0.015	0.020	0.000	0.001	0.002	0.003	0.014	0.013	0.000	0.004	0.004	0.000	0.000	0.004	0.000	0.000	0.000	0.001	0.12
	SE	0.012	0.012	0.010	0.010	0.011	0.010	0.009	0.008	0.009	0.009	0.009	0.010	0.008	0.007	0.008	0.008	0.007	0.007	0.006	0.007	0.005	0.005	
WHR	h^2_C	0.001	0.000	0.000	0.003	0.000	0.000	0.001	0.012	0.002	0.000	0.010	0.015	0.000	0.002	0.025	0.004	0.002	0.009	0.000	0.000	0.000	0.000	0.09
	SE	0.011	0.012	0.010	0.010	0.010	0.010	0.010	0.010	0.009	0.010	0.009	0.010	0.008	0.007	0.009	0.008	0.007	0.008	0.006	0.007	0.005	0.006	
Weight	h^2_C	0.008	0.035	0.000	0.002	0.016	0.026	0.000	0.004	0.004	0.009	0.005	0.010	0.001	0.003	0.014	0.002	0.000	0.014	0.000	0.000	0.000	0.012	0.16
	SE	0.012	0.013	0.010	0.010	0.011	0.011	0.009	0.009	0.009	0.010	0.009	0.009	0.008	0.007	0.008	0.008	0.007	0.008	0.006	0.007	0.005	0.006	
SBP0	h^2_C	0.009	0.020	0.011	0.010	0.025	0.011	0.021	0.013	0.000	0.000	0.013	0.016	0.006	0.005	0.005	0.005	0.006	0.013	0.003	0.014	0.000	0.016	0.22
	SE	0.012	0.012	0.011	0.010	0.011	0.010	0.011	0.010	0.009	0.009	0.009	0.010	0.008	0.008	0.008	0.008	0.007	0.008	0.006	0.008	0.005	0.006	
DBP0	h^2_C	0.012	0.024	0.006	0.002	0.013	0.017	0.002	0.008	0.011	0.002	0.000	0.000	0.006	0.004	0.004	0.000	0.015	0.008	0.009	0.014	0.000	0.017	0.17
	SE	0.012	0.012	0.011	0.010	0.010	0.010	0.010	0.010	0.009	0.009	0.009	0.009	0.008	0.008	0.007	0.008	0.008	0.008	0.006	0.007	0.006	0.007	
SBP	h^2_C	0.011	0.043	0.006	0.009	0.022	0.012	0.009	0.009	0.000	0.009	0.018	0.029	0.010	0.000	0.006	0.004	0.001	0.014	0.003	0.019	0.000	0.017	0.25
	SE	0.012	0.013	0.011	0.010	0.011	0.010	0.010	0.009	0.009	0.010	0.010	0.010	0.008	0.007	0.008	0.007	0.007	0.008	0.006	0.008	0.005	0.006	
DBP	h^2_C	0.024	0.027	0.010	0.017	0.024	0.012	0.000	0.002	0.000	0.013	0.016	0.010	0.003	0.000	0.010	0.005	0.000	0.007	0.004	0.017	0.000	0.015	0.22
	SE	0.013	0.012	0.011	0.011	0.011	0.010	0.010	0.009	0.009	0.010	0.009	0.009	0.008	0.008	0.008	0.008	0.007	0.007	0.006	0.008	0.005	0.006	
SUB	h^2_C	0.010	0.032	0.000	0.016	0.008	0.011	0.003	0.005	0.009	0.017	0.026	0.014	0.009	0.007	0.011	0.005	0.002	0.005	0.000	0.000	0.000	0.007	0.20
	SE	0.012	0.013	0.010	0.011	0.010	0.010	0.009	0.009	0.009	0.010	0.010	0.010	0.009	0.008	0.008	0.008	0.007	0.008	0.006	0.007	0.005	0.006	
SUP	h^2_C	0.001	0.017	0.000	0.000	0.004	0.002	0.000	0.006	0.000	0.000	0.032	0.009	0.000	0.004	0.002	0.000	0.001	0.005	0.000	0.000	0.000	0.005	0.09
	SE	0.012	0.013	0.010	0.011	0.010	0.010	0.009	0.009	0.009	0.010	0.010	0.010	0.009	0.008	0.008	0.008	0.007	0.008	0.006	0.007	0.005	0.006	
Pulse	h^2_C	0.029	0.009	0.000	0.002	0.000	0.019	0.022	0.000	0.000	0.000	0.000	0.017	0.000	0.009	0.003	0.000	0.005	0.002	0.007	0.006	0.003	0.001	0.13
	SE	0.012	0.011	0.011	0.010	0.010	0.010	0.010	0.009	0.009	0.010	0.009	0.010	0.008	0.008	0.008	0.008	0.007	0.007	0.006	0.007	0.005	0.005	
DS	h^2_C	0.011	0.006	0.017	0.000	0.011	0.009	0.011	0.001	0.000	0.011	0.018	0.025	0.000	0.000	0.000	0.001	0.000	0.000	0.004	0.004	0.003	0.005	0.14
	SE	0.012	0.012	0.012	0.010	0.011	0.010	0.010	0.009	0.009	0.011	0.010	0.011	0.008	0.008	0.008	0.008	0.007	0.008	0.006	0.007	0.006	0.006	
MS	h^2_C	0.004	0.009	0.000	0.000	0.000	0.009	0.009	0.000	0.026	0.002	0.012	0.005	0.000	0.005	0.008	0.009	0.000	0.003	0.000	0.000	0.000	0.003	0.10
	SE	0.013	0.012	0.011	0.010	0.011	0.010	0.010	0.009	0.011	0.010	0.010	0.010	0.009	0.008	0.008	0.008	0.008	0.008	0.006	0.007	0.006	0.005	
HDL	h^2_C	0.014	0.017	0.011	0.011	0.017	0.007	0.000	0.022	0.018	0.005	0.006	0.018	0.000	0.002	0.019	0.001	0.000	0.000	0.010	0.000	0.000	0.000	0.18

	SE	0.012	0.012	0.011	0.010	0.011	0.010	0.009	0.010	0.010	0.010	0.009	0.010	0.008	0.007	0.009	0.008	0.007	0.008	0.007	0.007	0.005	0.005	
TCHL	h^2_C	0.028	0.006	0.000	0.000	0.011	0.019	0.012	0.000	0.007	0.019	0.018	0.007	0.001	0.006	0.001	0.001	0.006	0.002	0.002	0.002	0.001	0.001	0.15
	SE	0.013	0.011	0.010	0.011	0.011	0.009	0.010	0.009	0.009	0.010	0.010	0.009	0.008	0.008	0.007	0.007	0.007	0.008	0.006	0.007	0.005	0.005	
TG	h^2_C	0.012	0.024	0.000	0.002	0.017	0.017	0.007	0.009	0.018	0.009	0.022	0.012	0.018	0.010	0.022	0.004	0.000	0.000	0.000	0.009	0.001	0.008	0.22
	SE	0.012	0.012	0.011	0.010	0.011	0.009	0.010	0.009	0.010	0.010	0.010	0.010	0.009	0.008	0.009	0.008	0.007	0.008	0.005	0.007	0.005	0.006	
LDL	h^2_C	0.023	0.015	0.001	0.000	0.011	0.011	0.013	0.001	0.000	0.014	0.019	0.003	0.000	0.003	0.000	0.001	0.005	0.000	0.007	0.004	0.001	0.000	0.13
	SE	0.013	0.012	0.011	0.010	0.011	0.010	0.010	0.009	0.009	0.010	0.010	0.009	0.008	0.008	0.008	0.007	0.007	0.008	0.007	0.007	0.005	0.006	
NONHDL	h^2_C	0.030	0.010	0.000	0.000	0.008	0.016	0.014	0.000	0.003	0.021	0.011	0.013	0.004	0.006	0.000	0.003	0.003	0.001	0.005	0.000	0.000	0.000	0.15
	SE	0.013	0.012	0.010	0.011	0.011	0.009	0.010	0.009	0.009	0.010	0.009	0.010	0.008	0.008	0.008	0.007	0.007	0.008	0.006	0.006	0.005	0.005	
THDL	h^2_C	0.026	0.020	0.001	0.000	0.005	0.005	0.003	0.015	0.008	0.015	0.000	0.027	0.005	0.005	0.012	0.005	0.000	0.000	0.013	0.000	0.000	0.000	0.16
	SE	0.013	0.012	0.010	0.010	0.010	0.009	0.009	0.010	0.009	0.010	0.008	0.010	0.008	0.008	0.008	0.008	0.007	0.008	0.007	0.007	0.005	0.005	
GLU0	h^2_C	0.011	0.000	0.000	0.023	0.000	0.001	0.019	0.005	0.014	0.006	0.000	0.000	0.000	0.001	0.006	0.000	0.002	0.000	0.011	0.015	0.004	0.004	0.12
	SE	0.012	0.012	0.010	0.011	0.010	0.009	0.010	0.009	0.010	0.010	0.009	0.009	0.008	0.008	0.008	0.008	0.007	0.007	0.007	0.008	0.006	0.005	
GLU60	h^2_C	0.000	0.015	0.000	0.002	0.004	0.003	0.012	0.000	0.001	0.022	0.006	0.000	0.020	0.000	0.011	0.000	0.007	0.000	0.003	0.001	0.000	0.000	0.11
	SE	0.012	0.012	0.011	0.010	0.011	0.010	0.010	0.010	0.009	0.011	0.009	0.009	0.009	0.007	0.009	0.008	0.007	0.008	0.006	0.007	0.006	0.005	
GLU120	h^2_C	0.000	0.000	0.000	0.018	0.000	0.007	0.024	0.001	0.000	0.008	0.005	0.004	0.015	0.001	0.013	0.014	0.000	0.000	0.018	0.002	0.010	0.004	0.14
	SE	0.012	0.012	0.012	0.011	0.010	0.010	0.011	0.009	0.009	0.010	0.009	0.010	0.009	0.007	0.009	0.008	0.007	0.008	0.007	0.007	0.006	0.005	
INS60	h^2_C	0.000	0.013	0.006	0.018	0.006	0.000	0.000	0.000	0.002	0.016	0.004	0.008	0.000	0.003	0.000	0.002	0.000	0.001	0.002	0.000	0.000	0.000	0.08
	SE	0.012	0.012	0.011	0.012	0.011	0.011	0.010	0.009	0.009	0.011	0.009	0.010	0.009	0.007	0.008	0.008	0.008	0.008	0.006	0.007	0.005	0.005	
INS120	h^2_C	0.006	0.035	0.020	0.009	0.016	0.015	0.012	0.000	0.000	0.004	0.002	0.002	0.000	0.004	0.000	0.005	0.000	0.003	0.002	0.000	0.002	0.001	0.14
	SE	0.012	0.013	0.012	0.011	0.011	0.011	0.010	0.010	0.010	0.010	0.009	0.009	0.009	0.008	0.008	0.008	0.007	0.008	0.005	0.007	0.006	0.005	
HBA1C	h^2_C	0.006	0.000	0.000	0.021	0.000	0.013	0.005	0.010	0.000	0.007	0.007	0.003	0.004	0.002	0.004	0.000	0.000	0.015	0.002	0.010	0.009	0.005	0.12
	SE	0.012	0.011	0.010	0.011	0.010	0.010	0.009	0.009	0.009	0.009	0.009	0.009	0.008	0.008	0.008	0.007	0.007	0.008	0.006	0.007	0.006	0.005	
WBC	h^2_C	0.009	0.018	0.005	0.000	0.010	0.018	0.002	0.021	0.000	0.021	0.000	0.011	0.000	0.011	0.006	0.009	0.002	0.000	0.016	0.000	0.004	0.003	0.16
	SE	0.012	0.012	0.010	0.009	0.010	0.011	0.010	0.010	0.009	0.010	0.009	0.009	0.008	0.008	0.008	0.008	0.007	0.007	0.007	0.007	0.006	0.005	
RBC	h^2_C	0.012	0.015	0.027	0.016	0.000	0.027	0.004	0.019	0.022	0.004	0.007	0.004	0.000	0.000	0.008	0.000	0.016	0.005	0.003	0.000	0.006	0.004	0.20
	SE	0.012	0.012	0.012	0.011	0.010	0.011	0.009	0.010	0.010	0.009	0.009	0.009	0.008	0.008	0.008	0.007	0.008	0.008	0.006	0.007	0.006	0.005	
SONA	h^2_C	0.000	0.010	0.008	0.017	0.001	0.000	0.001	0.004	0.003	0.000	0.000	0.008	0.000	0.000	0.003	0.009	0.000	0.000	0.009	0.000	0.000	0.000	0.07
	SE	0.011	0.011	0.011	0.011	0.010	0.009	0.009	0.009	0.009	0.010	0.009	0.010	0.008	0.007	0.008	0.008	0.007	0.008	0.006	0.007	0.005	0.005	
POTA	h^2_C	0.002	0.017	0.002	0.000	0.008	0.000	0.000	0.000	0.000	0.002	0.003	0.011	0.001	0.000	0.000	0.000	0.002	0.000	0.000	0.004	0.000	0.000	0.05
	SE	0.012	0.012	0.011	0.010	0.011	0.010	0.009	0.009	0.009	0.009	0.009	0.009	0.008	0.007	0.007	0.008	0.007	0.007	0.006	0.007	0.005	0.005	
CHL	h^2_C	0.014	0.000	0.008	0.000	0.021	0.000	0.002	0.000	0.002	0.000	0.027	0.009	0.000	0.003	0.010	0.010	0.012	0.000	0.006	0.000	0.007	0.001	0.13
	SE	0.012	0.011	0.011	0.010	0.011	0.009	0.010	0.009	0.009	0.009	0.010	0.010	0.008	0.007	0.008	0.008	0.008	0.007	0.006	0.007	0.006	0.005	

HB	h^2_C	0.004	0.019	0.002	0.004	0.000	0.000	0.002	0.004	0.012	0.002	0.014	0.001	0.000	0.002	0.000	0.000	0.013	0.000	0.000	0.000	0.000	0.002	0.08
	SE	0.011	0.012	0.011	0.010	0.010	0.009	0.009	0.009	0.010	0.009	0.009	0.009	0.008	0.007	0.008	0.008	0.008	0.008	0.006	0.007	0.005	0.005	
CRP	h^2_C	0.025	0.001	0.000	0.008	0.000	0.000	0.000	0.009	0.014	0.011	0.002	0.005	0.014	0.006	0.000	0.012	0.001	0.000	0.000	0.000	0.000	0.000	0.11
	SE	0.012	0.011	0.010	0.010	0.010	0.009	0.010	0.010	0.010	0.010	0.009	0.009	0.009	0.008	0.007	0.008	0.007	0.007	0.006	0.007	0.005	0.006	
RENIN	h^2_C	0.017	0.000	0.000	0.000	0.001	0.005	0.000	0.006	0.000	0.014	0.017	0.000	0.002	0.002	0.000	0.003	0.004	0.001	0.009	0.002	0.001	0.000	0.08
	SE	0.012	0.011	0.011	0.010	0.010	0.010	0.010	0.009	0.009	0.009	0.009	0.009	0.008	0.008	0.008	0.007	0.007	0.007	0.006	0.007	0.005	0.005	
PLAT	h^2_C	0.018	0.031	0.006	0.002	0.016	0.024	0.000	0.008	0.010	0.013	0.001	0.024	0.000	0.017	0.000	0.001	0.000	0.000	0.003	0.000	0.001	0.018	0.19
	SE	0.012	0.013	0.011	0.010	0.010	0.010	0.009	0.009	0.010	0.010	0.008	0.010	0.008	0.009	0.007	0.007	0.007	0.008	0.006	0.007	0.005	0.007	
SP1	h^2_C	0.000	0.014	0.000	0.010	0.002	0.020	0.000	0.016	0.022	0.028	0.018	0.016	0.000	0.010	0.004	0.020	0.009	0.001	0.005	0.012	0.005	0.000	0.21
	SE	0.012	0.012	0.010	0.011	0.010	0.010	0.010	0.010	0.010	0.011	0.010	0.010	0.008	0.008	0.008	0.009	0.008	0.007	0.006	0.007	0.005	0.005	
SP2	h^2_C	0.008	0.001	0.016	0.000	0.007	0.024	0.000	0.008	0.008	0.000	0.010	0.011	0.001	0.003	0.002	0.017	0.000	0.001	0.005	0.004	0.004	0.000	0.13
	SE	0.012	0.012	0.011	0.011	0.011	0.010	0.010	0.010	0.009	0.009	0.010	0.010	0.008	0.008	0.008	0.009	0.007	0.008	0.006	0.007	0.006	0.005	
SP3	h^2_C	0.026	0.016	0.000	0.016	0.000	0.019	0.003	0.006	0.017	0.006	0.000	0.000	0.003	0.000	0.000	0.001	0.013	0.002	0.000	0.001	0.012	0.001	0.14
	SE	0.012	0.012	0.011	0.011	0.010	0.010	0.010	0.009	0.010	0.010	0.009	0.009	0.008	0.008	0.007	0.008	0.008	0.008	0.006	0.007	0.006	0.005	
AST	h^2_C	0.003	0.026	0.003	0.004	0.000	0.001	0.008	0.003	0.000	0.002	0.000	0.014	0.000	0.000	0.001	0.002	0.001	0.002	0.000	0.004	0.000	0.000	0.07
	SE	0.012	0.012	0.010	0.010	0.010	0.009	0.010	0.009	0.009	0.009	0.009	0.010	0.009	0.007	0.007	0.007	0.007	0.007	0.006	0.007	0.006	0.005	
ALT	h^2_C	0.013	0.024	0.001	0.021	0.000	0.003	0.009	0.002	0.001	0.010	0.002	0.011	0.000	0.000	0.000	0.009	0.007	0.008	0.000	0.009	0.000	0.016	0.14
	SE	0.012	0.012	0.011	0.011	0.010	0.009	0.010	0.008	0.009	0.009	0.008	0.010	0.009	0.008	0.007	0.008	0.007	0.008	0.006	0.007	0.005	0.006	
RGTP	h^2_C	0.009	0.017	0.006	0.003	0.000	0.000	0.000	0.015	0.002	0.007	0.003	0.040	0.000	0.002	0.000	0.003	0.001	0.002	0.002	0.000	0.000	0.011	0.12
	SE	0.012	0.012	0.011	0.010	0.011	0.010	0.009	0.010	0.009	0.010	0.009	0.011	0.008	0.007	0.008	0.008	0.007	0.008	0.006	0.007	0.006	0.006	
BUN	h^2_C	0.007	0.000	0.027	0.000	0.006	0.003	0.000	0.009	0.000	0.016	0.000	0.000	0.000	0.000	0.006	0.000	0.019	0.011	0.001	0.006	0.002	0.000	0.11
	SE	0.012	0.011	0.012	0.010	0.010	0.010	0.009	0.009	0.008	0.010	0.008	0.009	0.009	0.007	0.008	0.008	0.008	0.008	0.006	0.007	0.005	0.005	
Creatine	h^2_C	0.000	0.006	0.010	0.007	0.000	0.000	0.002	0.000	0.000	0.000	0.004	0.000	0.005	0.000	0.006	0.000	0.003	0.004	0.000	0.000	0.002	0.000	0.05
	SE	0.012	0.012	0.011	0.010	0.010	0.009	0.009	0.010	0.009	0.010	0.009	0.009	0.008	0.008	0.008	0.008	0.007	0.008	0.006	0.007	0.005	0.005	
HCT	h^2_C	0.010	0.022	0.016	0.001	0.001	0.000	0.000	0.005	0.021	0.000	0.009	0.001	0.000	0.000	0.000	0.000	0.016	0.000	0.000	0.001	0.002	0.001	0.10
	SE	0.012	0.012	0.011	0.010	0.010	0.009	0.009	0.009	0.010	0.009	0.009	0.009	0.008	0.007	0.007	0.008	0.008	0.008	0.006	0.007	0.005	0.005	
pH	h^2_C	0.005	0.004	0.003	0.004	0.000	0.013	0.000	0.007	0.000	0.000	0.000	0.000	0.000	0.000	0.004	0.005	0.000	0.000	0.000	0.000	0.004	0.000	0.05
	SE	0.012	0.011	0.010	0.010	0.011	0.010	0.010	0.009	0.009	0.010	0.009	0.010	0.008	0.007	0.008	0.008	0.007	0.008	0.006	0.007	0.005	0.005	
SG	h^2_C	0.000	0.004	0.005	0.000	0.008	0.000	0.000	0.000	0.012	0.000	0.002	0.004	0.001	0.000	0.000	0.001	0.013	0.002	0.005	0.000	0.004	0.000	0.06
	SE	0.012	0.011	0.011	0.009	0.011	0.010	0.010	0.010	0.009	0.010	0.009	0.009	0.008	0.008	0.007	0.007	0.008	0.008	0.006	0.007	0.005	0.005	

Chr.: chromosome; L_C chromosome length; h^2_C: estimate of variance explained by all SNPs on a chromosome; SE: standard error of the estimate.
