# Supplementary material for: Ubiquitous Polygenicity of Human Complex Traits: Genome-Wide Analysis of 49 Traits in Koreans
Source: PLoS Genet. 2013 Mar 7;9(3):e1003355. doi: 10.1371/journal.pgen.1003355 (PMC3591292; doi:10.1371/journal.pgen.1003355)
Supplement: Table S4 — Estimates of the variance explained by all the genic and intergenic SNPs averaged across the 47 traits (all traits except INS0 and HOMA). A genic region is defined as ±0 kb, ±20 kb and ±50 kb of the 3′ and 5′ UTRs of a gene. (PDF) [file pgen.1003355.s012.pdf]

| | ± 0Kb | ± 20Kb | ± 50Kb |
|---|---|---|---|
| Coverage of genic regions | 36.1% | 49.2% | 58.9% |
| Estimated $h^2_{\mathrm{G_g}}$ | 0.0637 | 0.0889 | 0.0996 |
| SE | 0.0060 | 0.0066 | 0.0069 |
| Estimated $h^2_{\mathrm{G_i}}$ | 0.0655 | 0.0445 | 0.0341 |
| SE | 0.0068 | 0.0059 | 0.0052 |
| Expected $h^2_{\mathrm{G_g}}$ | 0.047 | 0.066 | 0.079 |
| Expected $h^2_{\mathrm{G_i}}$ | 0.083 | 0.068 | 0.055 |
| Goodness of fit test p-value | 1.6E-04 | 1.3E-07 | 5.4E-07 |

Note: The SE of averaged estimate was calculated as $SE = \frac{1}{t}\sqrt{\sum_{i=1} S_i^2}$ , where $S_i$ is the standard error of the estimate for each trait and $t$ is the effective number of independent traits with $t$ = 34.0, which was calculated by matrix spectral decomposition of the phenotypic correlation matrix [1].

## Reference


1. Li J, Ji L (2005) Adjusting multiple testing in multilocus analyses using the eigenvalues of a correlation matrix. Heredity (Edinb) 95: 221-227.
