# Supplementary material for: Ubiquitous Polygenicity of Human Complex Traits: Genome-Wide Analysis of 49 Traits in Koreans
Source: PLoS Genet. 2013 Mar 7;9(3):e1003355. doi: 10.1371/journal.pgen.1003355 (PMC3591292; doi:10.1371/journal.pgen.1003355)
Supplement: Table S5 — Numbers of genic and intergenic SNPs on each chromosome. (PDF) [file pgen.1003355.s013.pdf]

CHR	± 0kb		± 20kb		± 50kb	
	**Genic**	**Intergenic**	**Genic**	**Intergenic**	**Genic**	**Intergenic**
1	11,994	14,280	16,013	10,261	18,648	7,626
2	11,260	16,023	13,888	13,395	16,089	11,194
3	10,765	12,103	13,039	9,829	14,888	7,980
4	7,311	13,723	9,358	11,676	11,096	9,938
5	7,875	13,971	10,302	11,544	12,475	9,371
6	8,683	13,037	11,464	10,256	13,717	8,003
7	8,582	8,940	10,525	6,997	12,183	5,339
8	6,558	11,687	8,433	9,812	10,404	7,841
9	6,360	9,104	8,082	7,382	9497	5,967
10	8,526	10,563	10,537	8,552	12,251	6,838
11	7,029	10,261	9,936	7,354	11,494	5,796
12	6,609	10,121	9,103	7,627	10,859	5,871
13	4,147	8,471	5,216	7,402	6,318	6,300
14	3,970	6,502	5,434	5,038	6,481	3,991
15	4,298	5,285	5,468	4,115	6,475	3,108
16	4,446	5,511	5,708	4,249	6,631	3,326
17	3,670	3,625	5,020	2,275	5,693	1,602
18	3,417	6,358	4,456	5,319	5,281	4,494
19	2,161	2,054	3,342	873	3,668	547
20	3,644	4,591	4,886	3,349	5,613	2,622
21	1,945	2,838	2,569	2,214	2,982	1,801
22	2,241	1,723	2,858	1,106	3,158	806
