# Supplementary material for: Ubiquitous Polygenicity of Human Complex Traits: Genome-Wide Analysis of 49 Traits in Koreans
Source: PLoS Genet. 2013 Mar 7;9(3):e1003355. doi: 10.1371/journal.pgen.1003355 (PMC3591292; doi:10.1371/journal.pgen.1003355)
Supplement: Table S6 — Estimate of heritability from a pedigree analysis for 11 traits. Data and analysis are described in Text S1. (PDF) [file pgen.1003355.s014.pdf]

Group	Trait	n	h^2	SE
	Height	1264	0.744	0.048
**Obesity**	BMI	1264	0.478	0.057
	Waist	1264	0.358	0.057
	Hip	1263	0.473	0.058
	Weight	1264	0.480	0.058
**Lipids**	HDL	1264	0.739	0.048
	TCHL	1264	0.412	0.057
	TG	1264	0.441	0.060
	LDL	1264	0.399	0.057
**Liver Functions**	AST	1264	0.198	0.056
	ALT	1264	0.247	0.059
