# Supplementary material for: Ubiquitous Polygenicity of Human Complex Traits: Genome-Wide Analysis of 49 Traits in Koreans
Source: PLoS Genet. 2013 Mar 7;9(3):e1003355. doi: 10.1371/journal.pgen.1003355 (PMC3591292; doi:10.1371/journal.pgen.1003355)
Supplement: Table S7 — Estimates of variance explained by SNPs at the gene regions that are involved in insulin signaling pathway for 11 lipids and diabetes related traits. (PDF) [file pgen.1003355.s015.pdf]

Trait	Insulin signaling pathway			The rest of genome			Goodness of fit test
	**Estimated h_G^2**	**SE**	**Expected h_G^2**	**Estimated h_G^2**	**SE**	**Expected h_G^2**	***P-value***
HDL	0.000001	0.003	0.0008	0.176	0.041	0.175	0.812
TCHL	0.002754	0.003	0.0007	0.153	0.040	0.155	0.526
TG	0.000001	0.003	0.0010	0.218	0.041	0.217	0.744
LDL	0.000644	0.003	0.0006	0.134	0.041	0.134	0.991
NONHDL	0.002631	0.003	0.0007	0.155	0.040	0.156	0.548
THDL	0.000001	0.003	0.0007	0.164	0.040	0.163	0.800
GLU0	0.001103	0.003	0.0005	0.104	0.041	0.105	0.829
GLU60	0.000879	0.003	0.0005	0.099	0.043	0.100	0.885
GLU120	0.003874	0.003	0.0005	0.113	0.042	0.116	0.326
INS60	0.000001	0.003	0.0003	0.072	0.042	0.071	0.925
INS120	0.000001	0.003	0.0007	0.143	0.043	0.143	0.838
