# Supplementary material for: Ubiquitous Polygenicity of Human Complex Traits: Genome-Wide Analysis of 49 Traits in Koreans
Source: PLoS Genet. 2013 Mar 7;9(3):e1003355. doi: 10.1371/journal.pgen.1003355 (PMC3591292; doi:10.1371/journal.pgen.1003355)
Supplement: Text S1 — Difference between the estimates of variance explained by all SNPs in Europeans and in Koreans. (PDF) [file pgen.1003355.s016.pdf]

Text S1: Difference between the estimates of variance explained by all SNPs in Europeans and in Koreans

The estimate of h_G^2 (variance explained by all SNPs) for height in Koreans (h_G^2 = 0.316, SE = 0.042) was significantly smaller than that in Europeans (h_G^2 = 0.448, SE = 0.029) [1]. The estimate of h_G^2 for BMI in Koreans (h_G^2 = 0.165, SE = 0.029) was, however, not significantly different from that in Europeans (h_G^2 = 0.134, SE = 0.029). The difference for height was statistically significant, suggesting that it is likely to be a real difference. There could be two possible reasons 1) the heritability itself for height is smaller in Koreans than that in Europeans; 2) there is no difference in heritability for height between Koreans and Europeans but the SNP array (Affymetrix 5.0) used in this study is not as good as that used in [1] (Affymetrix 6.0) in tagging the causal variants. We therefore preformed additional analyses to test these two hypotheses.

We have got access to a dataset from a family study of 1758 Koreans genotyped on Affymetrix 6.0 array and with phenotype data available for 11 traits. After quality control (QC), 1,282 individuals and 520,484 SNPs were retained for analysis. We used GCTA to estimate the relatedness between individuals using all the autosomal SNPs and removed one of each pair of individual with an estimate of genetic relatedness > 0.8 (identical twins). We then used the SNP-derived pedigree structure to estimate the genetic variance in GCTA. The estimate of variance explained by all the SNPs from GCTA in family data is similar to the estimate of narrow-sense heritability (h^2) from a conventional pedigree analysis because the estimate is dominated by close-relatives. We chose GCTA to do the "pedigree analysis" simply because it is easy to use and it can recover some cryptic relatedness that were not documented in the pedigree record. As shown in the Table S5, the estimate of h^2 for height was 0.744 (SE = 0.048), which was not significantly (P = 0.243) different from that in Europeans (usually quoted as 0.8), and the estimate for BMI was 0.478 (SE = 0.057), which was also similar to the h^2 estimate for BMI in Europeans (usually quoted as 0.4 ~ 0.6). We then regressed the heritability estimated from the pedigree analysis in this studies against that from the literature in Europeans for the 11 traits (see figure below). The regression slope (0.83, SE = 0.39) was not significantly different from 1 (P = 0.67). All these results suggest that there is no significant difference in heritability between Koreans and Europeans for traits such as

height and BMI.

Figure. Heritability estimated from the pedigree analysis in Koreans against that from the literature in Europeans

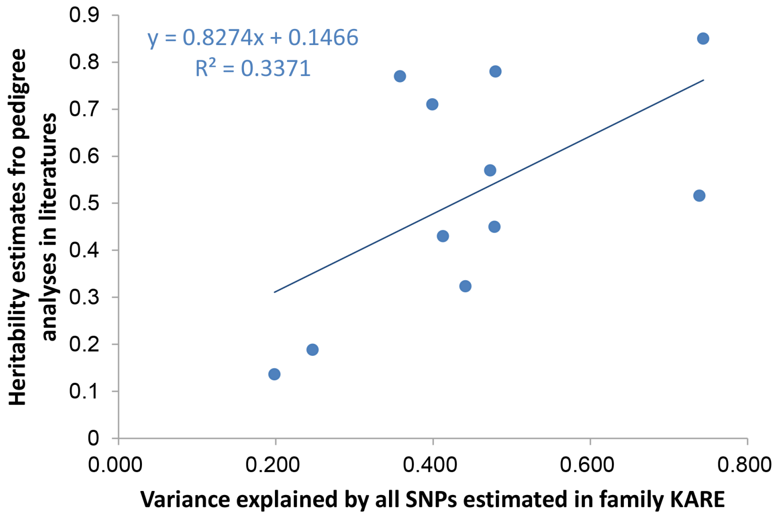

In addition, we have got access to the GWAS data of Europeans used in [1]. This dataset includes 11,586 unrelated European Americans selected from three population-based GWAS (the Health Professionals Follow-up Study (HPFS), the Nurses' Health Study (NHS) and the Atherosclerosis Risk in Communities (ARIC) study [2,3]). The samples were genotyped on Affymetrix 6.0 arrays. After QC, there were 565,040 autosomal SNPs. We included only the SNPs on Affymetrix 5.0 arrays (341,690 autosomal SNPs) in the analysis and estimated h_G^2 for height and BMI in these 11,586 unrelated European samples. The estimate of variance explained by all SNPs on Affymetrix 5.0 arrays was 0.394 (SE = 0.027) for height and 0.140 (SE = 0.026) for BMI. If we compare the estimate of h_G^2 from the Korean sample with that from the European sample, the difference is neither significant for height (P = 0.118) nor for BMI (P = 0.885).

In conclusion, there is no evidence suggesting that the heritability is different between Koreans and Europeans, and the difference in the variance explained by the same set of SNPs (SNPs on Affymetrix 5.0) between Koreans and Europeans is also not statistically significant.

Acknowledgements

HPFS and NHS data: Funding support for the GWAS of Gene and Environment

Initiatives in Type 2 Diabetes was provided through the NIH Genes, Environment and Health Initiative [GEI] (U01HG004399). The human subjects participating in the GWAS derive from The Nurses' Health Study and Health Professionals' Follow-up Study and these studies are supported by National Institutes of Health grants CA87969, CA55075, and DK58845. Assistance with phenotype harmonization and genotype cleaning, as well as with general study coordination, was provided by the Gene Environment Association Studies, GENEVA Coordinating Center (U01 HG004446). Assistance with data cleaning was provided by the National Center for Biotechnology Information. Funding support for genotyping, which was performed at the Broad Institute of MIT and Harvard, was provided by the NIH GEI (U01HG004424). The datasets used for the analyses described in this manuscript were obtained from dbGaP at [http://www.ncbi.nlm.nih.gov/sites/entrez?Db=gap] through dbGaP accession number [phs000091]

ARIC data: The Atherosclerosis Risk in Communities Study is carried out as a collaborative study supported by National Heart, Lung, and Blood Institute contracts (HHSN268201100005C, HHSN268201100006C, HHSN268201100007C, HHSN268201100008C, HHSN268201100009C, HHSN268201100010C, HHSN268201100011C, and HHSN268201100012C), R01HL087641, R01HL59367 and R01HL086694; National HumanGenome Research Institute contract U01HG004402; and National Institutes of Health contract HHSN268200625226C. The authors thank the staff and participants of the ARIC study for their important contributions. Infrastructure was partly supported by Grant Number UL1RR025005, a component of the National Institutes of Health and NIH Roadmap for Medical Research.

References

1. Yang J, Manolio TA, Pasquale LR, Boerwinkle E, Caporaso N, et al. (2011) Genome partitioning of genetic variation for complex traits using common SNPs. Nat Genet 43: 519-525.
2. Qi L, Cornelis MC, Kraft P, Stanya KJ, Linda Kao WH, et al. (2010) Genetic variants at 2q24 are associated with susceptibility to type 2 diabetes. Hum Mol Genet 19: 2706-2715.
3. Psaty BM, O'Donnell CJ, Gudnason V, Lunetta KL, Folsom AR, et al. (2009) Cohorts for Heart and Aging Research in Genomic Epidemiology (CHARGE) Consortium: Design of prospective meta-analyses of genome-wide association

studies from 5 cohorts. Circ Cardiovasc Genet 2: 73-80.
