# Supplementary material for: Ubiquitous Polygenicity of Human Complex Traits: Genome-Wide Analysis of 49 Traits in Koreans
Source: PLoS Genet. 2013 Mar 7;9(3):e1003355. doi: 10.1371/journal.pgen.1003355 (PMC3591292; doi:10.1371/journal.pgen.1003355)
Supplement: Text S2 — Difference in number of SNPs and MAF distribution between genic and intergenic SNPs. (PDF) [file pgen.1003355.s017.pdf]

Text S2: Difference in number of SNPs and MAF distribution between genic and intergenic SNPs

The results shown below are based on the genic definition of ±20Kb, with which the overall lengths of genic and intergenic regions are approximately equal.

1) Number of SNPs

We randomly sampled the same number of SNPs in the genic and intergenic regions on each chromosome. For example, if the number of genic and intergenic SNPs on one chromosome are M_g and M_i respectively with $M_g < M_i$, we will randomly sample M_g out of M_i SNPs in the intergenic regions, vice versa. We did so for each chromosome and re-estimated the variance explained by genic and intergenic SNPs on each chromosome. As shown in the figure below that the correlation between the estimate and DNA length is still stronger in the intergenic regions than that in the genic regions.

Figure. Estimate of the variance explained by genic (intergenic) SNPs on each chromosome averaged across all the traits against length of genic (intergenic) DNA when the numbers of the genic and intergenic SNPs on each chromosome are equal.

Genic SNPs **Intergenic SNPs**

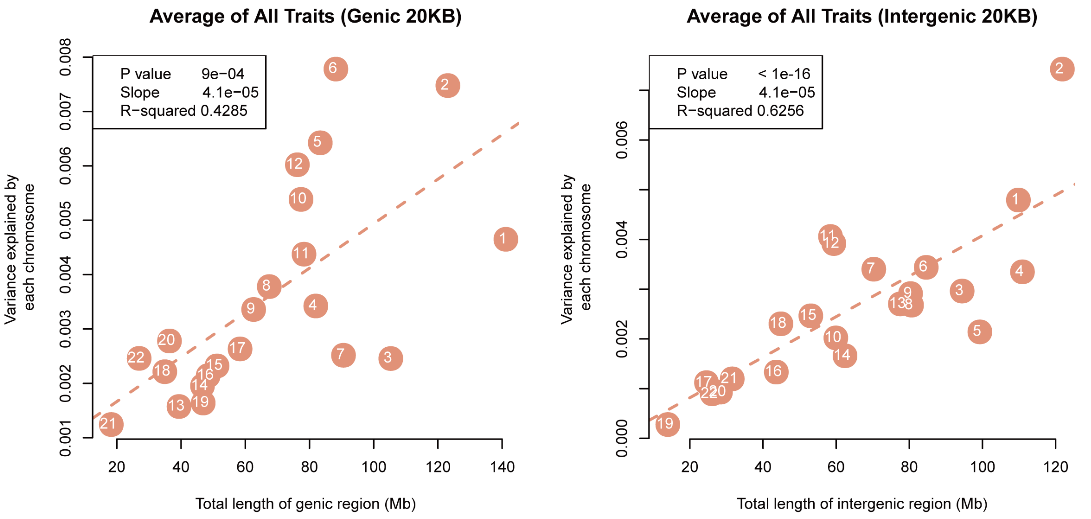

2) Minor allele frequency (MAF)

We show in the figure below that the mean MAF of intergenic SNPs (0.239) is

slightly larger than that of genic SNPs (0.236). However, it is difficult to test whether the difference is statistically significant or not due to linkage disequilibrium (SNPs are correlated).

Figure. Minor allele frequency distributions of the genic and intergenic SNPs.

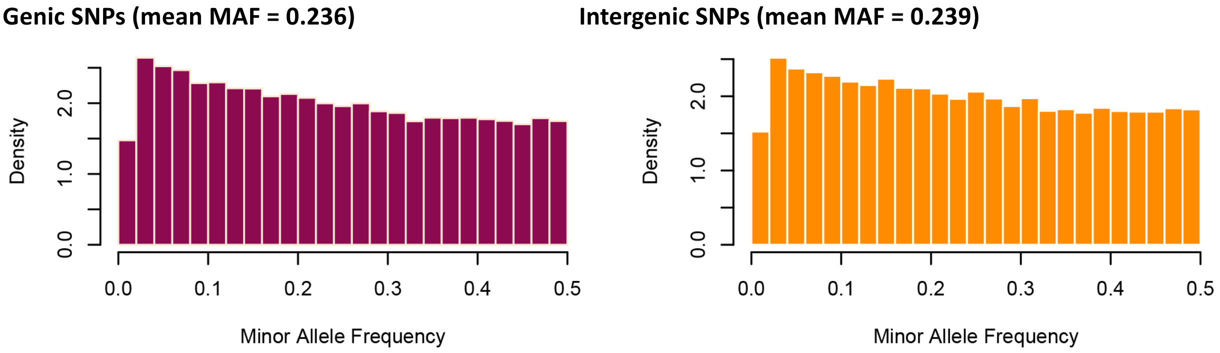

We then spit the whole MAF range into 50 bins (MAF bins: 0.01~0.02, 0.02~0.03, …, 0.49~0.5) and calculated the proportion of genic (intergenic) SNPs in each of these MAF bins. We then regressed the proportion of intergenic SNPs in each MAF bin against that of the genic SNPs (figure below). The regression slope (1.11, SE = 0.047) was significantly larger than one (P = 0.0181) suggesting the mean MAF of the intergenic SNPs was greater than that of the genic SNPs.

Figure. Proportion of genic SNPs vs. that of intergenic SNPs in each MAF bin

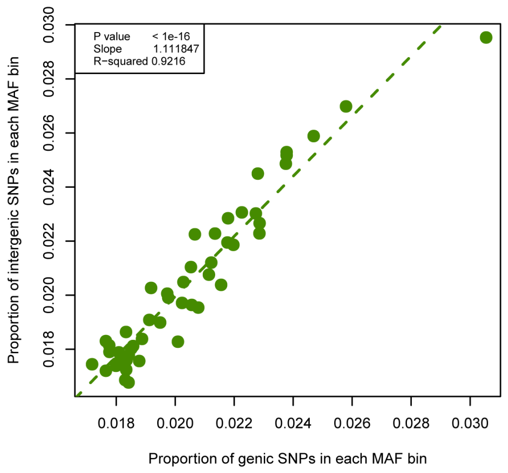

The recently published 1000 Genome Project paper [1] reported that there is an excess

of rare variants (MAF < 0.5%) at functional sites. It is unclear whether this implies anything about common variants on genotyping arrays in GWAS. Under the assumption that most mutations are deleterious, there would be relatively higher selection pressure on genetic variants at functional sites as compared with those at the other sites. We observed a consistent result in this study that the mean MAF of genic SNPs was slightly lower than that of intergenic SNPs.

We then preformed the chromosome partitioning analysis of genic and intergenic regions by sampling the same proportion of genic and intergenic in each of the 50 MAF bins so that the MAF distributions of the genic and intergenic were approximately the same. Let M_g and M_i be the total numbers of genic and intergenic SNPs respectively. Assuming there were x% (y%) of genic (intergenic) SNPs (the sum of x% or y% across all the MAF bins is one) in an MAF bin. When x% < y%, we sampled x%*M_i SNPs out of y%*M_i intergenic SNPs for that MAF bin, and when x% > y%, we sampled y%*M_g from x%*M_g genic SNPs for that MAF bin. By doing that, we created approximately the same MAF distribution for genic and intergenic SNPs. We re-ran the genic and intergenic partitioning analysis. As shown in the figure below, the correlation between variance explained and DNA length in the intergenic regions is still stronger than that in the genic regions, suggesting that the result was not driven by the difference in MAF distribution between genic and intergenic SNPs.

Figure. Estimates of the variance explained by all SNPs in genic (intergenic) regions averaged across all traits against length of genic (intergenic) DNA after adjusting for the difference in MAF distribution between genic and intergenic SNPs.

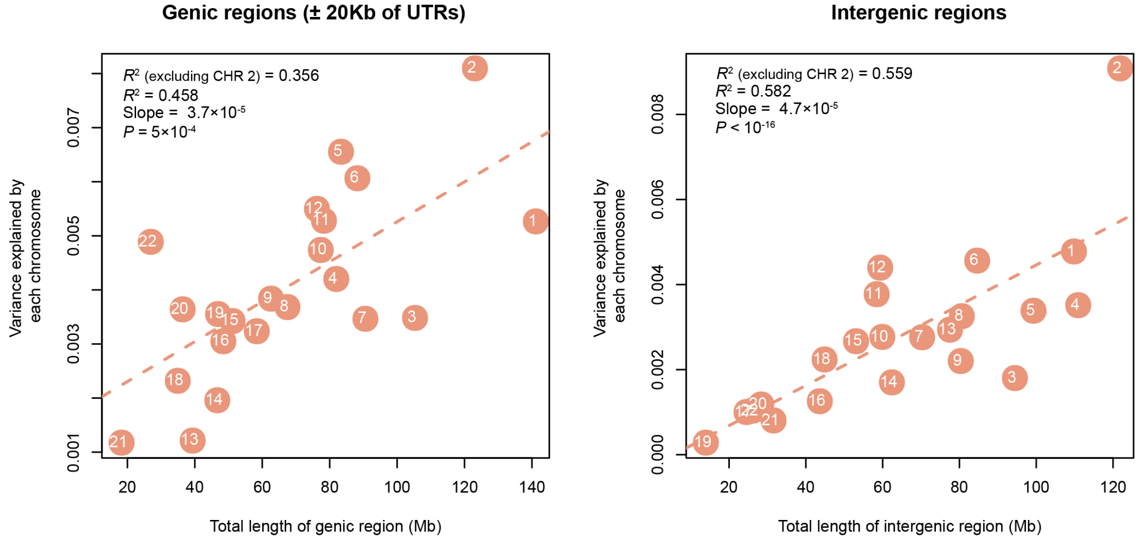

Reference

1. Abecasis GR, Auton A, Brooks LD, DePristo MA, Durbin RM, et al. (2012) An integrated map of genetic variation from 1,092 human genomes. Nature 491: 56-65.
